# Effects of two strains of *Lactobacillus* isolated from the feces of calves after fecal microbiota transplantation on growth performance, immune capacity, and intestinal barrier function of weaned calves

**DOI:** 10.3389/fmicb.2023.1249628

**Published:** 2023-08-31

**Authors:** Yuanyuan Li, Xin Li, Cunxi Nie, Yanyan Wu, Ruiqing Luo, Cheng Chen, Junli Niu, Wenju Zhang

**Affiliations:** ^1^College of Animal Science and Technology, Shihezi University, Shihezi, China; ^2^College of Life Sciences, Shihezi University, Shihezi, China; ^3^Xinjiang Tianshan Junken Animal Husbandry Co., Ltd.,Shihezi, China

**Keywords:** weaned calf, *Lactobacillus*, growth performance, immune capacity, intestinal barrier function

## Abstract

**Introduction:**

Weaning stress seriously affects the welfare of calves and causes huge economic losses to the cattle breeding industry. Probiotics play an important role in improving animal growth performance, enhancing immune function, and improving gut microbiota. The newly isolated strains of *Lactobacillus reuteri* L81 and *Lactobacillus johnsonii* L29 have shown potential as probiotics. Here, we studied the probiotic properties of these two strains on weaned calves.

**Methods:**

Forty calves were randomly assigned to four groups before weaning, with 10 calves in each group, control group (Ctrl group), *L. reuteri* L81 supplementation group (2 g per day per calf), *L. johnsonii* L29 supplementation group (2 g per day per calf), *L. reuteri* L81 and *L. johnsonii* L29 composite group (2 g per day per calf), and the effects of *Lactobacillus reuteri* L81 and *Lactobacillus johnsonii* L29 supplementation on growth performance, immune status, antioxidant capacity, and intestinal barrier function of weaned calves were evaluated.

**Results:**

The results showed that probiotics supplementation increased the average daily weight gain of calves after weaning, reduced weaning diarrhea index (*p* < 0.05), and increased serum IgA, IgM, and IgG levels (*p* < 0.05). *L. reuteri* L81 supplementation significantly decreased IL-6, increased IL-10 and superoxide dismutase (SOD) levels at 21 d after weaning (*p* < 0.05). Moreover, probiotics supplementation significantly decreased serum endotoxin (ET), diamine oxidase (DAO), and D-lactic acid (D-LA) levels at different time points (*p* < 0.05). In addition, supplementation with *L. reuteri* L81 significantly reduced the crypt depth and increased the ratio of villus height to crypt depth (*p* < 0.05) in the ileum, increased gene expression of tight junction protein *ZO-1*, *Claudin-1* and *Occludin* in jejunum and ileum mucosa, reduced the gene expression of *INF- γ* in ileum mucosa and *IL-8* in jejunum mucosa, and increased the abundance of beneficial bacteria, including *Bifidobacterium*, *Lactobacillus*, *Oscillospira*, etc.

**Discussion:**

verall, these results showed that the two strains isolated from cattle feces after low concentration fecal microbiota transplantation improved the growth performance, immune performance, antioxidant capacity, and intestinal barrier function of weaned calves, indicating their potential as supplements to alleviate weaning diarrhea in calves.

## 1. Introduction

Weaning is one of the most stressful periods in calf growth owing to the underdevelopment of the gastrointestinal system, incomplete intestinal microecosystem, low immune and antioxidant capacity, and weak digestion and absorption capacity of calves during the early weaning stage (Clark et al., 2016). Weaning stress, such as diarrhea, growth retardation, and even death, can occur as a result of dietary and psychological changes, which can seriously affect the welfare of calves and cause huge economic losses to the cattle breeding industry (Sweeney et al., 2010; Pempek et al., 2019). Antibiotics are widely used in animal husbandry to treat or prevent diarrhea and promote livestock growth (Brown et al., 2017); however, long-term antibiotics use can destroy the normal community structure of intestinal microorganisms and increase the presence of intestinal drug-resistant bacteria (Laxminarayan et al., 2013; Brand et al., 2017). Additionally, drug residues can pose potential problems, such as food safety issues, and threaten human health. Therefore, it is important to identify novel antibiotic substitutes capable of preventing or alleviating weaning stress in calves.

In 2001, the Food and Agriculture Organization of the United Nations and the World Health Organization (FAO/WHO) definition of probiotics -- “live microorganisms which when administered in adequate amounts confer a health benefit on the host” (Hill et al., 2014). Studies have shown that supplementation with probiotics plays an important role in improving animal production performance, reducing the incidence of diarrhea, enhancing immune function and antioxidant capacity, and improving intestinal microbial flora (Kelsey and Colpoys, 2018). Recently, there has been increasing research on effective antibiotics substitutes owing to health concerns associated with antibiotics use, and probiotics have been used as antibiotic substitutes in several countries. *Lactobacillus* plays a good role in improving intestinal epithelial barrier function, maintaining mucosal integrity, competing with pathogenic bacterial, and ameliorating intestinal mucosal damage (Wu et al., 2020). Our previous studies have shown that low-concentration fecal microbiota transplantation (LFMT) reduces the diarrhea incidence of weaned calves with Xinjiang yaks as the donor of fecal microbiota transplantation and Chinese Holstein calves as the recipient of fecal microbiota transplantation, and *Lactobacillus* was enriched in the intestine (Li et al., 2022). To develop more effective probiotics to alleviate weaning diarrhea in calves, we screened *Lactobacillus* in the feces of weaned calves after LFMT treatment in a previous study, and identified two potential probiotics for alleviating weaning diarrhea in calves, *Lactobacillus reuteri* L81 and *Lactobacillus johnsonii* L29.

*L. reuteri* and *L. johnsonii* have successfully been evaluated in humans and animals. *L. reuteri* probiotic preparations can improve gut function and enhance immunity, thereby promoting human health, enhancing pig growth, improving feed utilization efficiency, preventing diarrhea, and regulating the immune system in pigs (Hou et al., 2015). *L. reuteri* probiotics can replace antibiotics to promote the growth of weaned piglets and improve their intestinal health (Hong, 2016). *L. johnsonii* has been shown to prevent salmonella infection in weaned piglets, maintain metabolic homeostasis, enhance intestinal health in piglets (He et al., 2019), and regulate intestinal microbiota in mice. Based on these findings, the aim of this study was to examine the effects of these two strains on growth performance, diarrhea incidence, serum metabolites, immune performance, antioxidant capacity, and intestinal barrier function in early weaned calves. It is anticipated that the findings of this study would provide important evidence for implementing probiotic interventions and improving intestinal health in early weaned calves.

## 2. Materials and methods

### 2.1. Ethics statement

Animal experiment was conducted in accordance with the guidelines of the animal welfare and research institutions, and all procedures were approved by the Bioethics Committee of Shihezi University (License No.: A2021-32).

### 2.2. Animal management and diet

Forty Chinese Holstein calves of similar weight and birth date were randomly divided into four groups, with 10 calves in each group. To avoid cross-infection, all calves were placed on Calf Island. At 7 and 18 o’clock daily, the calves were fed twice with two equal volumes of milk in plastic buckets. At 50–56 d, the amount of milk consumed was 5 L/d. At 57–60 d, the calves were fed once daily, with the amount of milk reduced to 1 L/d, after weaning at 61 d, the feed amount was decreased to 0. The milk was produced on the same farm and pasteurized at 60°C before use. Starter concentrate was provided by Xinjiang Urumqi Zhengda Feed Co., Ltd. (Urumqi, China). All calves can drink water, take starter and *Arrhenatherum elatius* freely after weaning. The nutritional compositions of the diets are listed in Supplementary Table S1.

### 2.3. Experimental design, sample collection, and analysis

At 50–60 d of age, group 1 was fed normally, without any treatment, so it was the control group (Ctrl group). Group 2 was given *L. reuteri* L81 every day (2 g per day per calf, R group). Group 3 was given *L. johnsonii* L29 every day (2 g per day per calf, J group). Group 4 was given *L. reuteri* L81 and *L. johnsonii* L29 compound bacteria (2 g per day per calf, R + J group). The concentration of *Lactobacillus* used in the study was adjusted to 1 × 10^8^ CFU/g. To ensure that *Lactobacillus* was fully consumed by the calves, freeze-dried *Lactobacillus* powder was dissolved in milk for morning feeding. This study was conducted at the Zhenxing Farm of the Tianshan Military Reclamation Company in Shihezi, Xinjiang, China, from May to July 2022.

### 2.4. Growth performance, diarrhea rate and diarrhea index

The body weight of each calf was measured before morning feeding on the day of weaning (T0), and at 7 days (T1), 14 days (T2), and 21 days (T3) after weaning. Feed intake was recorded daily, and the average daily gain (ADG), average daily feed intake (ADFI), and feed conversion rate (F/G) were calculated.

Fecal samples from the calves were scored according to the diarrhea index scoring method. The diarrhea index was scored as follows: normal feces (solid), 0 points; wet feces (semi-solid),1 point; mild diarrhea (pasty feces), 2 points; and severe diarrhea (watery stool), 3 points. The number of days and heads of calves with diarrhea during the trial period were recorded to determine the diarrhea index and diarrhea rate statistics (Mcguirk, 2008). The following formulas were used to calculate the diarrhea rate and index for each group of calves,

Diarrhea index=∑fecal scores/number of calves×test days

Diarrhea rate (%) = number of calves with diarrhea per group during the trial period × diarrhea days / (trial days × number of calves per group) × 100%.

### 2.5. Sample collection

Blood samples were collected from the jugular veins of six calves in each group before morning feeding at 7 (T1), 14 (T2), and 21 d (T3) after weaning in a 10 mL vacuum blood collection tube without anticoagulant. The blood samples were centrifugated at 3000 × *g* for 15 min, and the supernatant was stored in a 2 mL tube at −20°C until further analysis.

Calves were weaned at 60 d of age. At 21 d after weaning, five calves from the control (Ctrl group) and *L. reuteri* supplementation groups (R group) were anesthetized *via* intramuscular injection of 4% pentobarbital sodium solution at a dose of 40 mg/kg body weight and euthanized by jugular venous bleeding. The abdominal cavity was quickly opened, and the contents of the duodenum, jejunum, and ileum were collected and divided them into 2 mL and 10 mL sterile enzyme free tubes. The contents of the tubes were snap-frozen in liquid nitrogen. After flushing out the contents of the intestinal canal with phosphate buffer solution (pH 7.4), the intestinal mucosa was carefully cleaned of fatty tissues and stored in a 2 mL sterile enzyme free EP tube for rapid freezing. Intestinal contents and intestinal mucosa were transported to the laboratory in a frozen tube and stored at −80°C until further analysis.

In addition, the middle segments of duodenum, jejunum and ileum were selected. After the contents of the intestinal tube were flushed out with phosphate buffer solution with pH = 7.4, they were quickly placed in 4% paraformaldehyde fixative at 4°C for 48 h for tissue sectioning.

### 2.6. Determination of biochemical indicators

Serum total protein (TP), albumin (ALB), glucose (GLU), triglyceride (TG), and blood urea nitrogen (BUN) levels were determined using a biochemical detection kit from Nanjing Jiancheng Biotechnology Research Institute, according to the manufacturer’s instructions.

### 2.7. Antioxidant indicators, immune indicators, cytokines and intestinal permeability indicators in serum

Superoxide dismutase (SOD) and malondialdehyde (MDA) levels were determined by spectrophotometry using corresponding kits (Nanjing Jiancheng Biotechnology Research Institute, Nanjing, China), according to the manufacturer’s instructions.

IgA, IgG, IgM, IL-2, IL-6, IL-10, TNF- α, endotoxin (ET), diamine oxidase (DAO), and D-lactic acid (D-LA) contents were determined using ELISA kits (Shanghai Enzymes Biotechnology Co., Ltd.), according to the manufacturer’s instructions.

### 2.8. Intestinal tissue morphology

Each intestinal segment was sectioned by haematoxylin eosin (HE) staining method described by Hu J. et al. (2018) for intestinal histomorphology measurement. The main steps include fixation, dehydration, immersing the tissue block in xylene until completely transparent, placing the transparent tissue in paraffin, embedding, slicing, 37°C water bath development, drying in a 37°C oven, dewaxing, ethanol gradient elution, hematoxylin staining, eosin staining, ethanol gradient dehydration, sealing, and air drying. And its morphology and structure were observed at 10× under an optical microscope. The villus height and recess depth of small intestine were measured, and the ratio of villus height to recess depth was calculated.

### 2.9. Detection of intestinal tight junction protein and cytokine gene expression

The frozen duodenum, jejunum and ileum mucosa were taken out and placed in a autoclaved mortar, and the liquid nitrogen was added to grind and homogenize. Then, an appropriate amount of powder samples were taken, and total RNA was extracted from the tissue mucosa with TRIzol reagent. The total RNA was reverse transcribed and cDNA was synthesized according to the instructions of the reverse transcription kit. Primer 3 was used for primer design, and the primers were detailed in Supplementary Table S2. The primers were synthesized by Shanghai Shenggong Biotechnology Co., Ltd. The mRNA expression levels of intestinal tight junction proteins *Claudin-1*, *Occuludin* and *ZO-1*, intestinal mucosal cytokines *TLR4*, INF-γ, *TGF-β1*, *IL-8* and *IL-2* were detected by real-time fluorescent quantitative PCR with specific primers.

### 2.10. Determination of enzyme activity in the small intestine

After the calves were slaughtered, the intestinal contents were taken and placed in a clean centrifuge tube. They were transported to the laboratory and their pH value were immediately measured using a pH meter.

0.5 g of duodenal, jejunal, and ileal contents were diluted with physiological saline at a ratio of 1:9, followed by homogenization in an ice bath and centrifugation at 4500 × *g* for 20 min to obtain supernatant for enzyme activity assay. Amylase, trypsin, lipase, lactase, and Na-K-ATPase activities were determined using the respective ELISA kits (Shanghai Enzymes Biotechnology Co., Ltd.), according to the manufacturer’s instructions.

### 2.11. DNA extraction, PCR amplification of 16SrRNA, and sequencing

DNA extraction, genome library construction, and intestinal microbial sequencing were performed by Shanghai Parsenor Biotechnology Co. Ltd. Briefly, total DNA was extracted from the intestinal contents, and the V3-V4 hypervariable region of bacterial 16S rRNA was amplified using the universal primers 338F (5′-ACTCCTACGGGGAGCA-3′) and 806R (5′- GGACTACHVGGGTWTCTAAT-3′). After purification, all amplified products were sequenced using the Illumina MiSeq platform. The PCR amplification reaction system contained 5 μL of buffer (5 ×), 0.25 μL of fast pfu DNA polymerase (5 U/μL), 2 μL (2.5 mM) of dNTPs, 1 μL (10 uM) forward primer and 1 μL reverse primer, 1 μL of DNA template, and 14.75 μL of ddH_2_O. The PCR conditions were as follows: initial denaturation at 98°C for 5 min, followed by 25 cycles at 98°C for 30 s, annealing at 53°C for 30 s, and extension at 72°C for 45 s, and final extension at 72°C of 5 min. PCR amplicons were purified using Vazyme VAHTSTM DNA clean beads (Vazyme, Nanjing, China) and quantified using a Quant-iT PicoGreen dsDNA Assay Kit (Invitrogen, Carlsbad, CA, USA). After the individual quantification step, amplicons were pooled in equal amounts, and paired-end sequencing (2 × 250 bp) was performed by Shanghai Personal Biotechnology Co., Ltd. (Shanghai, China) on the Illumina MiSeq platform using the MiSeq Reagent Kit v3.

Bioinformatics analysis of the intestinal bacteria was conducted using QIIME2 2019.4, with slight modifications based on the official manual. Microbial α-diversity was evaluated using Shannon, Simpson, Chao1, and Coverage indices, while β-diversity was analyzed using nonmetric multidimensional scale. Shared and unique ASVs between samples or groups were visualized using the “VennDiam” function in R software. LEfSe analysis was used to compare the relative abundance of the intestinal microbiota between the control and *L. reuteri* L81 supplemented groups at the phylum and genus levels.

### 2.12. Statistics and analysis

One-way analysis of variance was used to analyze multiple sets of data, and the results were expressed as mean and standard deviation. Calculate the gene expression data of the sample using the 2^-ΔΔ^Ct method. The differences of intestinal villus height, crypt depth and the ratio of villus height to crypt depth, intestinal tight junction protein and cytokine gene expression were performed using *t*-tests. The data were expressed by mean value and standard error, means were considered statistically significant at *p* < 0.05. All statistical analyses were performed using SPSS 26.0 software (SPSS, Inc., Chicago, IL, United States).

## 3. Results

### 3.1. Effects of *Lactobacillus reuteri* L81 and *Lactobacillus johnsonii* L29 on growth performance, diarrhea incidence, and diarrhea index of weaned calves

**Figure 1:**
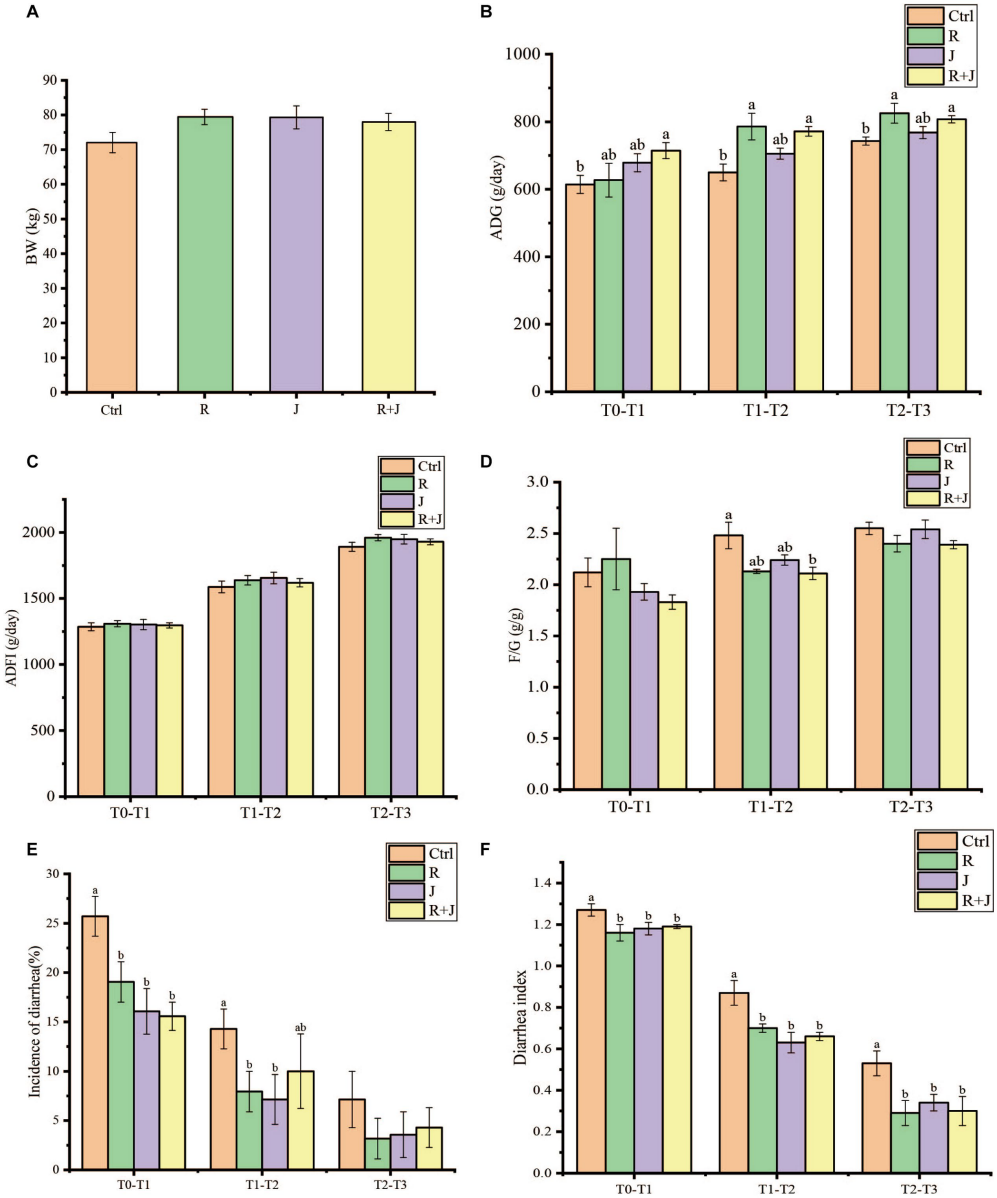
Effect of *L. reuteri* L81 and *L. johnsonii* L29 on growth performance, diarrhea incidence and diarrhea index of weaned calves. **(A)** Initial calf weight (BW). **(B)** Average daily weight gain (ADG). **(C)** Average daily food intake (ADFI). **(D)** Material weight ratio (F/G). **(E)** Incidence of calves diarrhea. **(F)** Calves diarrhea index. At the same time point, if there was no letter or the same letter was marked, the difference was not significant (*p* > 0.05), and different lower case letters indicate significant difference (*p* < 0.05). The same below.

There was no significant difference (*p* > 0.05) in the initial weight of the calves among the experimental groups (Figure 1). In contrast, calves in the R + J group had significantly higher ADG at T0–T1, while those in the R and R + J groups had significantly higher ADG at T1–T2 and T2–T3 compared with the Ctrl group (*p* < 0.05). However, there was no significant difference in ADG between the J and Ctrl groups, and no significant difference between R, J and R + J groups. Additionally, there was no significant difference in ADFI between the experimental groups at all time points. Moreover, F/G ratio was significantly lower in the R + J group than in the Ctrl group at T1–T2 (*p* < 0.05); however, there was no significant difference between the groups at T0–T1 and T2–T3.

Calf diarrhea incidence was significantly higher in the Ctrl group than in the R, J, and R + J groups at T0–T1 and T2–T3 (*p* < 0.05) (Figure 1); however, there was no significant difference between the lactobacilli supplementation groups. Similarly, diarrhea incidence was significantly lower in the R and J groups at T1–T2 (*p* < 0.05); however, there was no significant difference in diarrhea incidence between the R + J and Ctrl groups at the time point. Additionally, average fecal score was significantly higher in the Ctrl group than in the other treatment groups at all time points. However, there was no significant difference in average fecal score between the probiotics supplementation groups, indicating that the selected lactic acid bacteria are capable of alleviating and preventing calf diarrhea.

### 3.2. Effects of *Lactobacillus reuteri* L81 and *Lactobacillus johnsonii* L29 on serum biochemical indicators

Serum TP level was significantly higher (*p* < 0.05) in the R, J, and R + J groups than in the Ctrl group at T3 (Table 1); however, there was no significant difference (*p* > 0.05) in serum TP level between the probiotics supplementation groups. Similarly, serum GLB level was significantly higher (*p* < 0.05) in the R and R + J groups than in the Ctrl group at T3. Additionally, serum BUN level was significantly higher in the R group than in the Ctrl group at T1 and T2; however, there was no significant difference in serum BUM levels between the probiotics and Ctrl groups at T3. Moreover, there was no significant difference in GLU levels between the R and Ctrl groups. Interestingly, serum GLU level was significantly higher in the J group than in the Ctrl group at T2, and significantly higher in the R + J group than in the R and J groups at T3.

**Table 1:** Effect of *L. reuteri* L81 and *L. johnsonii* L29 on serum biochemical indexes of weaned calves.

Item	Time	Experimental Treatments				*p*-value
		Ctrl	R	J	R + J	
TP (g/L)	T1	59.3 ± 2.3	54.3 ± 2.0	57.3 ± 2.7	55.8 ± 4.7	0.702
	T2	54.7 ± 1.9	57.0 ± 1.8	59.5 ± 1.4	55.9 ± 1.7	0.289
	T3	55.3 ± 1.2^b^	65.9 ± 2.9^a^	65.0 ± 3.24^a^	61.1 ± 0.82^a^	0.026
ALB (g/L)	T1	33.9 ± 0.6	32.2 ± 0.7	33.3 ± 0.6	31.9 ± 0.5	0.470
	T2	33.3 ± 0.9	32.8 ± 0.8	35.6 ± 1.4	33.7 ± 0.5	0.223
	T3	34.3 ± 1.5	32.1 ± 1.0	34.3 ± 1.3	35.4 ± 0.6	0.283
GLB (g/L)	T1	25.5 ± 1.8	22.1 ± 1.7	24.0 ± 3.2	23.9 ± 3.6	0.854
	T2	21.4 ± 2.5	24.2 ± 1.0	23.9 ± 0.6	22.2 ± 1.2	0.348
	T3	20.9 ± 1.5^b^	33.8 ± 3.3^a^	30.7 ± 4.5^ab^	25.7 ± 0.8^a^	0.042
BUN (mmol/L)	T1	2.94 ± 0.20^b^	3.93 ± 0.29^a^	3.09 ± 0.24^ab^	2.69 ± 0.11^b^	0.031
	T2	3.09 ± 0.16^b^	4.73 ± 0.47^a^	4.37 ± 0.62^a^	3.27 ± 0.29^b^	0.009
	T3	3.80 ± 0.22	3.48 ± 0.14	4.23 ± 0.15	4.30 ± 0.39	0.117
GLU (mmol/L)	T1	5.01 ± 1.00	7.32 ± 0.59	3.75 ± 0.41	4.24 ± 1.27	0.052
	T2	3.21 ± 0.10^b^	2.97 ± 0.51^b^	6.01 ± 0.88^a^	3.98 ± 0.26^b^	0.003
	T3	5.47 ± 0.30^ab^	4.53 ± 0.50^bc^	3.37 ± 0.31^c^	5.94 ± 0.28^a^	0.001
TG (Triglyceride, mmol/L)	T1	0.33 ± 0.07	0.29 ± 0.02	0.34 ± 0.04	0.15 ± 0.05	0.08
	T2	0.25 ± 0.05	0.30 ± 0.05	0.35 ± 0.05	0.20 ± 0.03	0.159
	T3	0.30 ± 0.07	0.19 ± 0.04	0.23 ± 0.04	0.41 ± 0.09	0.123

TP, Total Protein; ALB, Albumin; GLB, Globulin; BUN, Blood Urea Nitrogen; GLU, Glucose; TG, Triglyceride. 7 days, 14 days and 21 days after weaning of calves were expressed as T1, T2 and T3, respectively. Ctrl, control group; R, *L. reuteri* L81 supplementation group; J, *L. johnsonii* L29 supplementation group; R + J, *L. reuteri* L81 and *L. johnsonii* L29 composite group. In the same line, marked with different letters means significant difference between groups (*p* < 0.05).

### 3.3. Effects of *Lactobacillus reuteri* L81 and *Lactobacillus johnsonii* L29 on immune indicators, antioxidant indicators, cytokines, and intestinal permeability indicators

To evaluate the role of *L. reuteri* L81 and *L. johnsonii* L29 in the immune system, the serum immunoglobulins levels of the weaned calves were measured after probiotics supplementation (Table 2). Compared with the Ctrl group, serum IgA and IgM levels were significantly higher (*p* < 0.05) in the *Lactobacillus*-supplemented group at T1, T2, and T3. Specifically, calves in the J group had significantly higher serum IgM levels than those in the other groups at T1 and T2; however, there was no significant difference in IgM levels among the *Lactobacillus* supplementation groups at T3. Serum IgG level was significantly higher in the R group than in the Ctrl group at T2 and T3. Additionally, calves in the J group had significantly higher serum IgG levels than those in the other groups at T1. However, there was no significant difference (*p* > 0.05) in serum TNF-α level between the Ctrl and probiotic groups at T1, T2, and T3. Serum IL-10 concentration was significantly higher in the R group than in the Ctrl group at T1 and T3. Similarly, serum IL-10 concentration was significantly higher (*p* < 0.05) in the J group than in other groups at T2. Although there was no significant difference in serum IL-6 concentration between the groups at T1 and T2, the R group had significant lower serum IL-6 concentration at T3.

**Table 2:** Effect of *L. reuteri* L81 and *L. johnsonii* L29 on immune indexes, antioxidant indexes, biochemical indexes of intestinal barrier function in serum of weaned calves.

Item	Time	Experimental treatments				*p*-value
		Ctrl	R	J	R + J	
IgA (μg/mL)	T1	1465.07 ± 105.12^b^	2041.49 ± 164.81^a^	2309.75 ± 121.75^a^	2097.46 ± 134.07^a^	0.002
	T2	1808.00 ± 137.94^b^	2489.38 ± 127.90^a^	2501.16 ± 163.09^a^	2127.21 ± 106.90^ab^	0.004
	T3	1603.59 ± 180.81^c^	2211.86 ± 39.52^b^	2340.06 ± 64.91^ab^	2630.13 ± 179.51^a^	<0.001
IgM (μg/mL)	T1	784.67 ± 71.28^c^	1066.59 ± 88.55^b^	1586.91 ± 56.48^a^	1064.44 ± 32.15^b^	0.033
	T2	711.12 ± 26.46^d^	988.51 ± 58.42^c^	1679.83 ± 7.03^a^	1297.32 ± 81.62^b^	<0.001
	T3	1052.49 ± 86.54^b^	1526.73 ± 70.21^a^	1446.32 ± 75.19^a^	1480.35 ± 104.98^a^	0.002
IgG (mg/mL)	T1	3.21 ± 0.47^b^	3.96 ± 0.24^b^	5.2 ± 0.31^a^	4.54 ± 0.36^b^	0.004
	T2	2.89 ± 0.36^b^	5.93 ± 0.32^a^	5.71 ± 0.33^a^	5.78 ± 0.32^a^	<0.001
	T3	3.81 ± 0.24^b^	5.39 ± 0.39^a^	5.92 ± 0.26^a^	5.85 ± 0.39^a^	<0.001
MDA (nmol/mL)	T1	6.02 ± 0.36	4.64 ± 0.38	5.18 ± 0.31	5.27 ± 0.52	0.131
	T2	5.06 ± 0.28	4.85 ± 0.39	4.51 ± 0.29	5.57 ± 0.52	0.292
	T3	4.39 ± 0.31	3.97 ± 0.21	4.06 ± 0.36	4.73 ± 0.44	0.409
SOD (U /mL)	T1	12.01 ± 0.42^b^	13.33 ± 0.51^ab^	14.10 ± 0.35^a^	13.02 ± 0.31^ab^	0.017
	T2	13.28 ± 0.41	13.72 ± 0.10	13.61 ± 0.33	12.73 ± 0.35	0.167
	T3	11.72 ± 0.17^b^	12.78 ± 0.27^a^	12.92 ± 0.45^a^	13.17 ± 0.44^a^	0.049
TNF-α (pg/mL)	T1	141.14 ± 10.03	114.80 ± 6.62	136.02 ± 6.27	119.75 ± 12.58	0.141
	T2	113.12 ± 8.85	114.80 ± 8.80	103.58 ± 9.20	111.89 ± 10.47	0.798
	T3	126.70 ± 3.33	139.26 ± 7.35	125.59 ± 3.78	131.18 ± 15.11	0.603
IL-6 (pg/mL)	T1	89.02 ± 14.95	77.13 ± 4.04	88.41 ± 5.02	100.99 ± 9.87	0.460
	T2	85.06 ± 5.14	97.09 ± 3.32	84.39 ± 4.63	98.22 ± 4.25	0.056
	T3	121.93 ± 4.60^a^	86.92 ± 12.42^b^	115.53 ± 3.42^ab^	107.27 ± 10.80^ab^	0.046
IL-10 (pg/mL)	T1	44.12 ± 1.65^c^	57.53 ± 2.19^b^	70.15 ± 0.19^a^	58.41 ± 4.51^b^	<0.001
	T2	50.03 ± 2.50^b^	56.52 ± 3.30^b^	75.17 ± 3.94^a^	56.67 ± 2.02^b^	<0.001
	T3	49.34 ± 0.85^c^	62.37 ± 3.80^b^	75.96 ± 3.80^a^	60.62 ± 4.04^b^	0.007
ET (EU/mL)	T1	14.45 ± 0.75	12.37 ± 0.57	12.27 ± 0.81	12.58 ± 0.76	0.149
	T2	15.22 ± 0.21^a^	12.47 ± 0.57^b^	9.63 ± 0.91^c^	10.47 ± 0.58^c^	<0.001
	T3	13.15 ± 0.76^a^	10.93 ± 1.18^ab^	8.22 ± 0.53^b^	10.03 ± 0.99^b^	0.007
DAO (ng/mL)	T1	164.41 ± 2.61^a^	151.69 ± 1.73^b^	113.03 ± 9.68^c^	134.84 ± 10.73^bc^	0.001
	T2	154.39 ± 5.79^a^	108.30 ± 3.70^b^	104.84 ± 4.45^b^	121.81 ± 6.96^b^	<0.001
	T3	141.23 ± 1.90^a^	98.44 ± 11.72^b^	66.54 ± 4.55^c^	104.08 ± 12.11^b^	<0.001
D-LA (μmol/mL)	T1	0.56 ± 0.04^a^	0.52 ± 0.01^b^	0.44 ± 0.01^c^	0.55 ± 0.03^b^	0.006
	T2	0.56 ± 0.02^a^	0.44 ± 0.01^b^	0.46 ± 0.01^b^	0.49 ± 0.03^ab^	0.001
	T3	0.53 ± 0.01^a^	0.45 ± 0.04^ab^	0.35 ± 0.03^b^	0.46 ± 0.03^b^	0.005

IgA, Immunoglobulin A; IgM, Immunoglobulin M; IgG, Immunoglobulin G; MDA, Malondialdehyde; SOD, Superoxide Dismutase; TNF-α, Tumor necrosis factor-α; IL-6, Interleukin-6; IL-10, Interleukin-10; ET, Endotoxins; DAO, Diamine oxidase; D-LA, D-Lactic acid. In the same line, marked with different letters means significant difference between groups (*p* < 0.05).

Calves in the J group had significantly higher serum SOD concentration than those in the Ctrl group at T1 (*p* < 0.05); however, there was no significant difference in serum SOD level between the J, R, and R + J groups at the time point. Calves in the probiotic groups (R, J, and R + J) had significantly higher (*p* < 0.05) serum SOD concentrations than those in the Ctrl group at T3.

Furthermore, the effects of the probiotics on intestinal barrier function in weaned calves were examined by determination the serum levels of intestinal permeability indicators (Table 2). There was no significant difference in serum ET levels between the *Lactobacillus* supplementation groups (R, J, and R + J) and the Ctrl group at T1; however, serum DAO and D-LA levels were significantly lower in the *Lactobacillus* supplementation groups than in the Ctrl group (*p* < 0.05). Calves in the *Lactobacillus* supplementation groups had significantly lower serum ET and DAO levels than those in the Ctrl group at T2; additionally, serum D-LA level was significant lower in the R and J groups than in the Ctrl group at T2 (*p* < 0.05). At T3, serum ET and D-LA levels were significantly lower in the J and R + J groups than in the Ctrl group (*p* < 0.05), meanwhile there was no significant difference between the Ctrl and R groups. *Lactobacillus* supplementation significantly decreased serum DAO levels compared with the Ctrl group, with the lowest level observed in the J group.

### 3.4. Effect of *Lactobacillus reuteri* L81 on intestinal morphology of weaned calves

The effect of *L. reuteri* L81 on the intestinal tissue morphology of weaned calves is shown in Table 3. The results showed that the villus height and the ratio of villus height to crypt depth of each small intestinal segment in the R group of Holstein weaned calves were higher than those in the Ctrl group, and the crypt depth was lower than that in the Ctrl group. The crypt depth of ileal in the R group was significantly reduced compared to the Ctrl group, and the villus height / crypt depth was significantly increased (*p* < 0.05).

**Table 3:** Effect of *L. reuteri* L81 on small intestinal morphology development of weaned calves.

Item	Ctrl	R	SEM	*p*-value
Villus height, μm				
Duodenum	543.63	600.82	18.06	0.117
Jejunum	485.39	549.77	19.71	0.103
Ileum	409.56	419.32	9.51	0.654
Crypt depth, μm				
Duodenum	308.24	298.96	9.34	0.656
Jejunum	325.31	315.91	8.70	0.628
Ileum	320.58^a^	217.39^b^	22.98	0.008
Villus height/ Crypt depth				
Duodenum	1.77	2.01	0.07	0.075
Jejunum	1.50	1.74	0.08	0.129
Ileum	1.28^b^	2.01^a^	0.19	0.041

### 3.5. Effects of *Lactobacillus reuteri* L81 on the expression of intestinal tight junction protein and cytokines related genes in weaned calves

**Figure 2:**
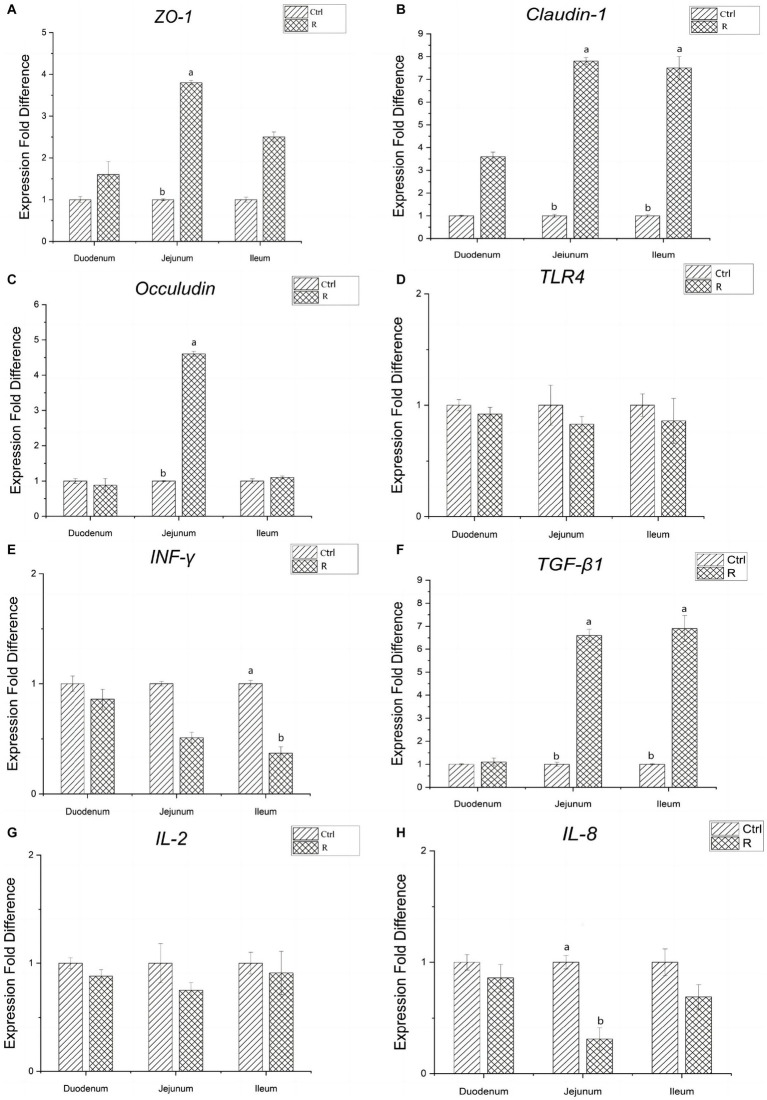
Effects of *L. reuteri* L81 on the expression of intestinal tight junction protein and cytokines related genes in weaned calves. **(A)** Relative gene expression of *ZO-1*. **(B)** Relative gene expression of *Claudin-1.* **(C)** Relative gene expression of *Occludin.* **(D)** Relative gene expression of *TLR4.* **(E)** Relative gene expression of *INF-γ.* **(F)** Relative gene expression of *TGF-β1.* **(G)** Relative gene expression of *IL-2.* **(H)** Relative gene expression of *IL-8*.

It can be seen from Figure 2 that, compared with the Ctrl group, the gene expression of tight junction protein in duodenum of group R has no significant change. The gene related expression of tight junction protein *Claudin-1*, *Occludin* and *ZO-1* in jejunum was significantly increased (*p* < 0.05). The relative expression of *Claudin-1* gene in the ileum increased significantly (*p* < 0.05). There was no significant difference in the relative expression of *TLR4* and *IL-2* genes between the R group and the Ctrl group (*p* > 0.05). The expression of *TGF-β1* in the jejunum and ileum of group R was significantly increased, and the expression of *IL-8* in the jejunum and *INF-γ* in the ileum were significantly reduced (*p* < 0.05).

### 3.6. Effect of *Lactobacillus reuteri* L81 on pH value and enzyme activity in small intestine

**Figure 3:**
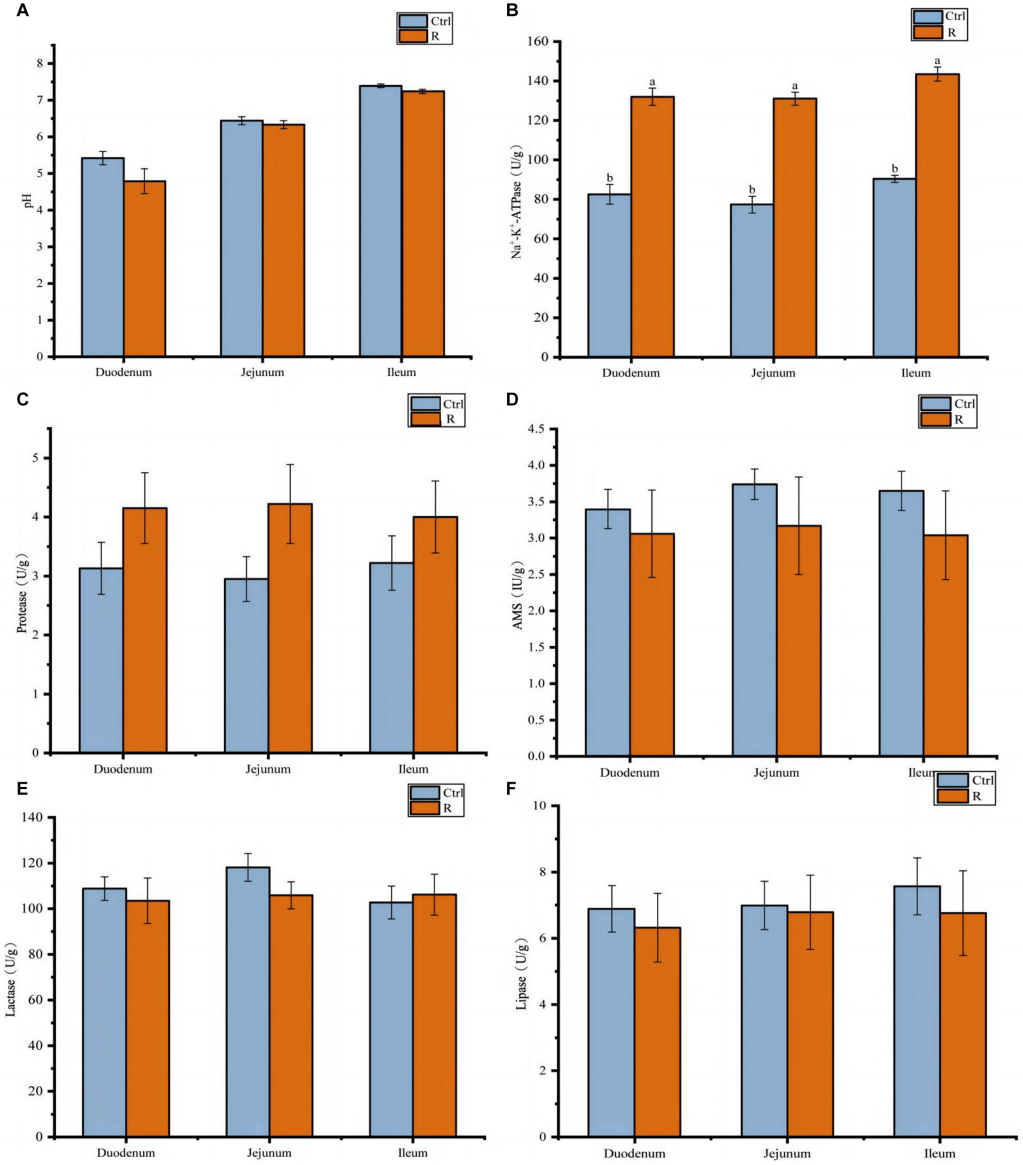
Effect of *L. reuteri* L81 on pH value and digestive enzyme activities of intestinal contents in weaned calves. **(A)** pH value. **(B)** Na^+^-K^+^-ATPase. **(C)** Protease. **(D)** AMS. **(E)** Lactase. **(F)** Lipase.

The effects of *L. reuteri* L81 on intestinal content pH and enzyme activity in the duodenum, jejunum, and ileum were shown in Figure 3. It can be seen from Figure 3A that supplementation of *L. reuteri* L81 before weaning had no significant effect on intestinal pH after weaning. However, Na^+^-K^+^-ATPase activity in the duodenum, jejunum, and ileum was significantly higher in the R group than in the Ctrl group (*p* < 0.05). Compared with the Ctrl group, the supplementation of *L. reuteri* L81 had no significant effect on the activities of protease, amylase, lipase and lactase.

### 3.7. Quality evaluation of sequencing data of intestinal microbial communities

**Figure 4:**
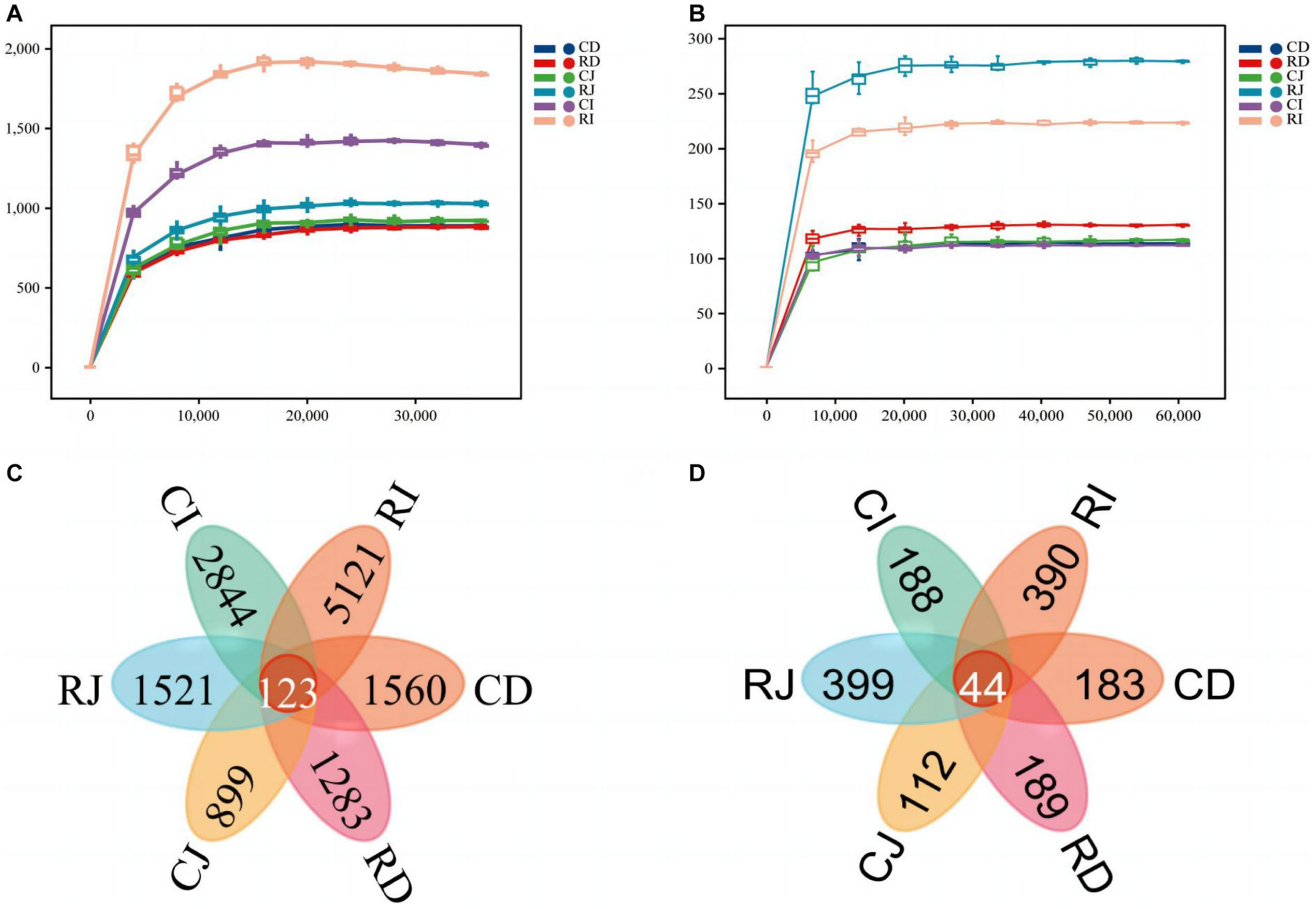
Bacterial and fungal rarefaction curve and venn diagrams of ASVs in different intestinal segments of different sample groups. **(A)** Bacterial rarefaction curve based on Chao index. **(B)** Fungal rarefaction curve based on Chao index. The X-axis is the sequencing depth, and the Y-axis is the median value and boxplot of Chao index calculated for 10 times. **(C)** Venn diagram of bacteria ASVs. **(D)** Fungus ASVs Venn diagram. CD, ctrl group duodenum; CJ, ctrl group jejunum; CI, ctrl group ileum; RD, *L. reuteri* L81 group duodenum; RJ, *L. reuteri* L81 group jejunum; RI, *L. reuteri* L81 group ileum.

Sequencing of the V3–V4 regions of the 16S rRNA gene in DNA samples generated a total of 2,211,341 raw reads. After double-ended quality control, splicing, noise removal, chimerism removal, and singleton removal of raw reads, 1,308,705 valid sequences were obtained for subsequent analysis. The fungal ITS1-2 region was sequenced and 2,512,684 valid sequences were obtained for subsequent analysis. The sparse curves of all samples were nearly flat, indicating that the sequencing amount of each sample was sufficient to cover most of the bacterial and fungal communities and that the sequencing results reflected the composition and diversity of the bacterial and fungal communities in the samples (Figure 4). To assess the commonality and uniqueness of microorganisms in different intestinal segments, the number of ASVs common to different intestinal segments among the groups was analyzed using a Venn diagram. In the Ctrl and R groups, there were 123 ASVs for bacteria and 44 ASVs for fungi in the different intestinal segments, and each group of samples had unique ASVs. The number of unique ASVs in the ileum was higher than those in the duodenum and jejunum. Compared with the Ctrl group, a higher number of unique ASVs were observed in the jejunum and ileum of calves in the R group.

### 3.8. Effect of *Lactobacillus reuteri* L81 on the diversity of intestinal microbiota in weaned calves

**Figure 5:**
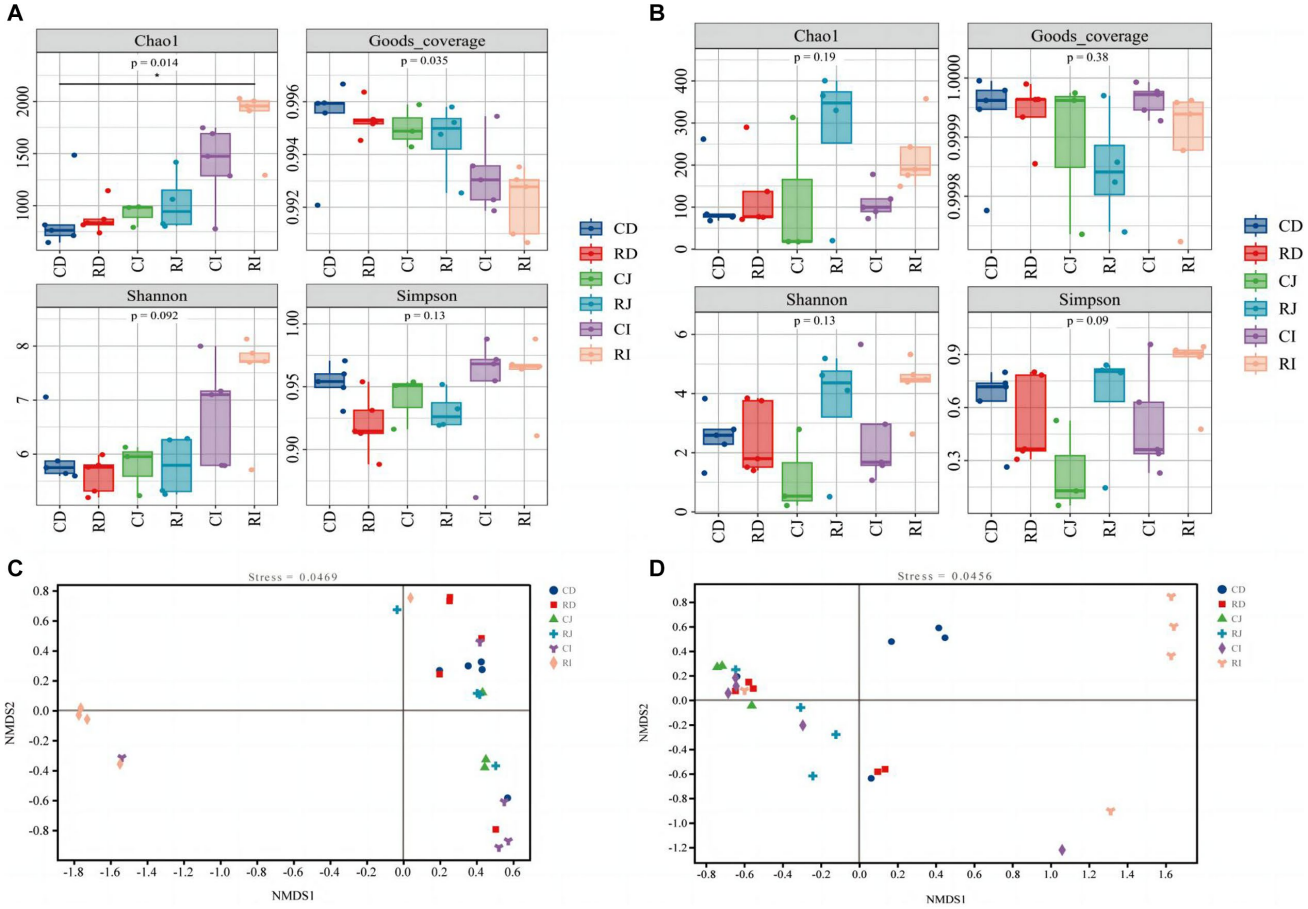
Effect of *L. reuteri* L81 on diversity of intestinal microbiota in weaned calves. **(A)** Bacterial α diversity index. **(B)** Fungal α diversity index. **(C)** NMDS diagram of bacteria. **(D)** NMDS diagram of fungal. 0.01 < *p* < 0.05 in the figure indicates significant difference, marked with ^*^; *p* < 0.01 indicates extremely significant difference, marked with ^**^. CD, ctrl group duodenum; CJ, ctrl group jejunum; CI, ctrl group ileum; RD, *L. reuteri* L81 group duodenum; RJ, *L. reuteri* L81 group jejunum; RI, *L. reuteri* L81 group ileum.

Furthermore, the effect of *L. reuteri* L81 supplementation on duodenal, jejunal, and ileal microbiota diversity were examined (Figure 5). The Good’s coverage index of each group of samples was above 0.99, indicating that the sequencing data sufficiently reflected the composition and distribution of the intestinal microbiota. Compared with the control group, the Chao1 and Shannon indices of bacteria and fungi in the different intestinal segments showed an upward trend in the R group; however, there was no significant difference in Simpson index between the groups.

A non-metric multidimensional scale (NMDS) analysis of the composition of microbes in the duodenum, jejunum, and ileum of weaned calves was performed based on Bray Curtis distance (Figure 5). Intestinal microbiota in the same intestinal tract formed distinct clusters among individuals; however, there were individual differences between the groups, with the R group showing a significant separation of the ileal microbiota from the duodenal and jejunal microbiota.

### 3.9. Effect of *Lactobacillus reuteri* L81 on the structure of intestinal microbiota in weaned calves

**Figure 6:**
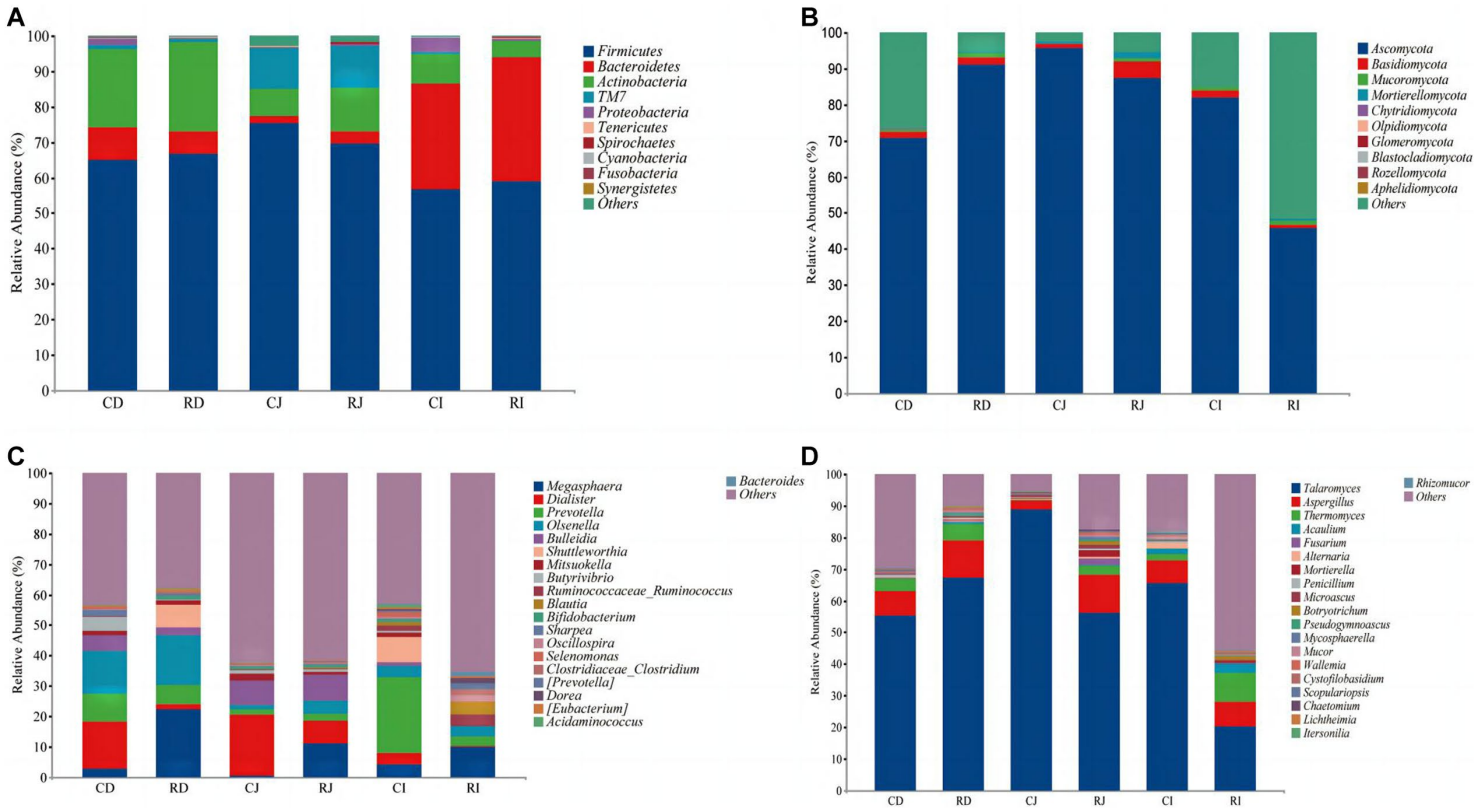
Effects of *L. reuteri* L81 on the intestinal microbiota composition of weaned calves at the level of phylum and genus. **(A)** The microbiota composition of bacteria at the phylum level. **(B)** The microbiota composition of fungal at the phylum level. **(C)** The microbiota composition of bacteria at the genus level. **(D)** The microbiota composition of fungal at the genus level. CD, ctrl group duodenum; CJ, ctrl group jejunum; CI, ctrl group ileum; RD, *L. reuteri* L81 group duodenum; RJ, *L. reuteri* L81 group jejunum; RI, *L. reuteri* L81 group ileum.

At the phylum level, the dominant bacterial microbiota in the duodenum, jejunum, and ileum were *Firmicutes*, followed by *Actinobacteria* and *Bacteroides*. The dominant bacteria in the duodenum and jejunum were *Firmicutes* and *Actinobacteria*, while those in the ileum were *Firmicutes* and *Bacteroides* (Figure 6). The relative abundance of *Bacteroidetes* was significantly higher in the ileum than in the duodenum and jejunum. Compared with the Ctrl group, *L. reuteri* L81 supplementation significantly decreased the relative abundance of *Proteobacteria* in the ileum of the calves. *Ascomycota* was the dominant fungal phylum in the duodenum, jejunum, and ileum, followed by *Basidiomycota* and *Mucoromycota*. *Ascomycota* fluctuated between groups, and the relative abundance of *Ascomycota* in the ileum of the R group was significantly lower than that in the other experimental groups.

At the genus level, *Megaspaera*, *Dialister*, *Prevotella*, *Olsenella*, and *Bulleidia* were the predominant bacteria in the duodenum and jejunum. *Megaspaera*, *Prevotella,* and *Dialister* were the dominant genera in the ileum, followed by *Ruminococcaceae* (*Ruminococcus*) and *Blautia*. However, *L. reuteri* L81 supplementation increased the relative abundances of *Ruminoccaceae*_*Ruminococcus*, *Blautia*, and *Oscillospira* in the ileum and decreased the relative abundance of *Dialister* in the duodenum, jejunum, and ileum. *Talaromyces* and *Aspergillus* were the dominant fungal genera in the duodenum, jejunum, and ileum, followed by *Thermomyces*.

**Figure 7:**
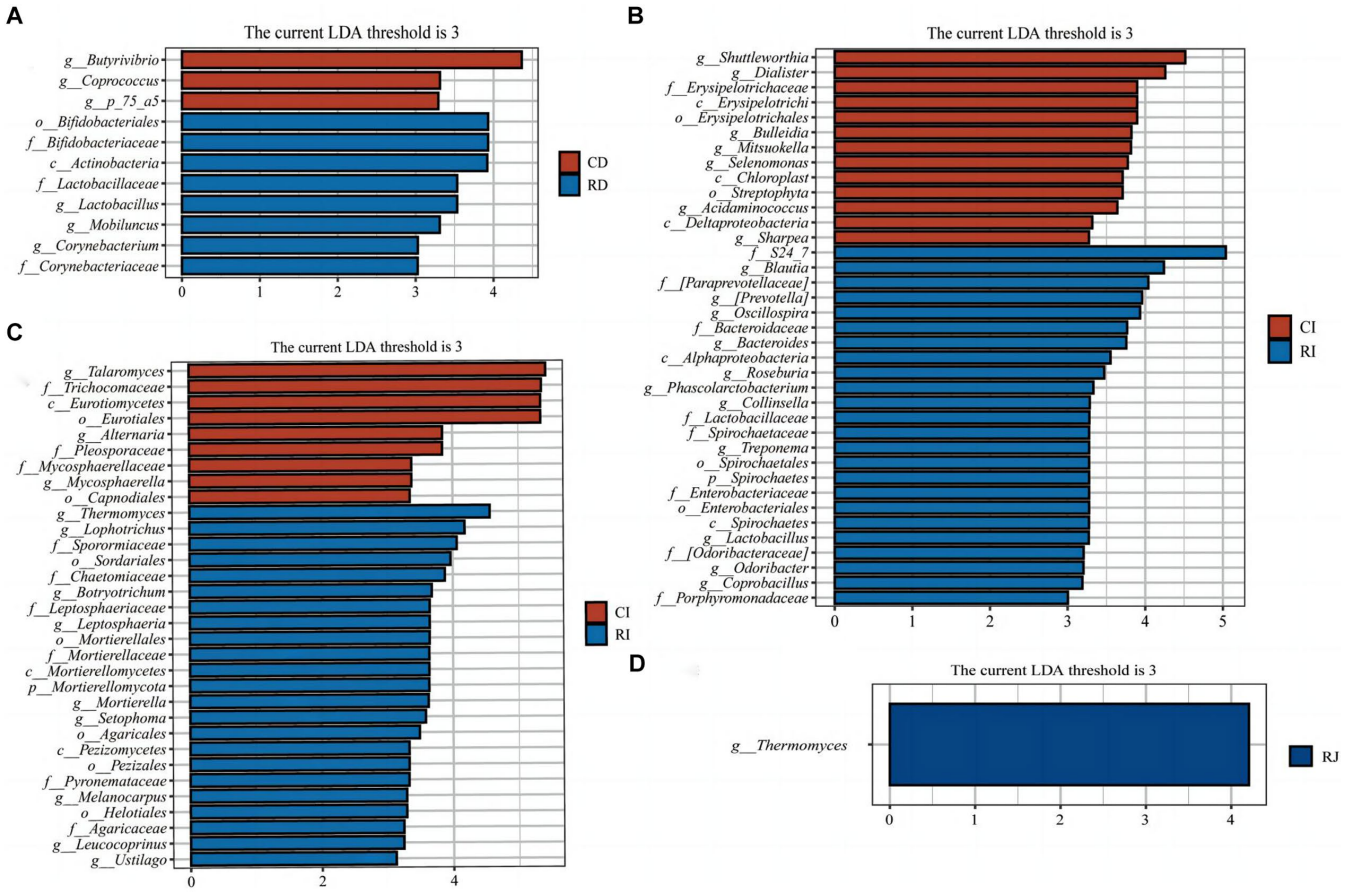
LEfSe analysis of intestinal microbiota in weaned calves. **(A)** Histograms of LEfSe analysis of duodenal bacteria in the Ctrl group and *L. reuteri* L81 group. **(B)** Histograms of LEfSe analysis of ileal bacteria in the Ctrl group and *L. reuteri* L81 group. **(C)** Histogram of LEfSe analysis of ileal fungi in the Ctrl group and *L. reuteri* L81 group. **(D)** Histogram of LEfSe analysis of jejunum fungi in the Ctrl group and *L. reuteri* L81 group. The ordinate of the histogram is the classification group with significant difference between groups, and the abscissa is the bar chart showing the logarithmic score of LDA of each classification group. The longer the length is, the more significant the difference of taxon is. The color of the bar chart indicates the sample group with the highest abundance corresponding to the taxon.

LEfSe analysis was performed to further identify microbial compositional differences in intestinal segments between the Ctrl and R groups (Figure 7). *L. reuteri* L81 supplementation enriched *Bifidobacteriales*, *Lactobacillus*, *Mobilucus* and *Corynebacterium* in the duodenum; *Thermomyces* in the jejunum; and *Blautia*, *Collinsella*, *Oscillospira*, *Roseburia*, *Coprobacillus*, *Odoribacter*, *Lactobacillus*, *Thermogenes*, *Lophotrichus*, and *Botryotrruchum* in the ileum.

## 4. Discussion

During weaning, calves are prone to stress, which can affect their health and growth performance, and lead to stunted growth and development. Probiotics play a positive role in promoting animal health, improving host immunity, combating pathogenic bacteria, regulating intestinal microecology, and improving enhancing intestinal epithelial barrier function (Aoudia et al., 2016; David et al., 2016; Kelsey and Colpoys, 2018).

### 4.1. Growth performance, diarrhea incidence, and diarrhea index

In the present study, supplementation with *L. reuteri* L81and *L. reuteri* L81 + *L. johnsonii* L29 improved ADG in wean calves compared with the Ctrl group at T1–T2 and T2–T3. Similarly, *L. reuteri* supplementation has been shown to significantly improve the ADG of piglets (Wang et al., 2020); in contrast, some studies have shown that the addition of *L. reuteri* or the combination of *L. reuteri* and other probiotics had no significant effect on ADG in weaned piglets (Dell’Anno et al., 2021), which could be attributed to differences in probiotic strains, supplementation amount, or combinations. Additionally, supplementation with *L. reuteri* L81 + *L. johnsonii* L29 significantly reduced the F/G ratio at T1–T2. Previous studies have shown that the supplementation of *L. reuteri* or probiotics before weaning can improve the growth of calves.

Early intervention with probiotics has been reported to prevent calf diarrhea, reduce calf mortality, and improve calf health by promoting gastrointestinal health and maintaining intestinal barrier integrity in pre-weaning calves (Liu et al., 2015). A previous study showed that *L. reuteri* and *L. johnsonii* exhibited potent antibacterial activity against pathogens that commonly cause bovine diarrhea (Fan et al., 2021). *L. reuteri* has a strong ability to adhere to the intestinal mucosa, which can improve the distribution of intestinal microbiota and inhibit colonization by harmful bacteria (Deng et al., 2020). Moreover, *L. johnsonii* supplementation has been shown to improve intestinal environment, immunity, and disease resistance in piglets (Xin et al., 2019). In the present study, supplementation with *L. reuteri* L81and *L. johnsonii* L29 reduced diarrhea incidence at T0–T1 and T1–T2 after weaning, which was consistent with previous findings. Although there was no significant difference in diarrhea rate between the groups at T2–T3, *Lactobacillus* supplementation significantly reduced the diarrhea index of calves. Overall, these results indicated that the probiotics were effective in preventing weaning diarrhea in calves when administered individually or in combinations.

### 4.2. Serum biochemical indicators

ALB has several physiological functions, such as maintaining normal colloidal osmotic pressure in blood vessels, transporting various metabolic substances, and regulating transported substances. GLB is primarily involved in immune regulation, while BUN is a product of protein metabolism and an important indicator of renal function. In the present study, serum TP and GLB levels were significantly higher in the R group than in the Ctrl group at T3, indicating that *L. reuteri* L81 improved immune performance in weaned calves. Similarly, calves in the R group had significantly higher BUN levels than those in the Ctrl group at T1 and T2, indicating that amino acid metabolism was elevated in weaned calves and that accumulated nitrogen could not be discharged in a timely manner (Lu et al., 2021; Han et al., 2022). GLU is the main indicator of glucose metabolism in the body and is a direct energy source for the body’s activities. In the present study, *L. reuteri* L81 and *L. johnsonii* L29 supplementation had different effects on serum GLU levels, indicating that different microecological preparations have different effects on energy and lipid metabolism in weaned calves.

### 4.3. Immune function, antioxidant capacity, and intestinal permeability

Serum IgG, IgM, and IgA levels reflect the functional state of humoral immunity (Kotnik and Zaletel, 2011; Panahi et al., 2020; Tan et al., 2021). Some studies have shown that *L. johnsonii* supplementation can improve serum IgG levels in sows (Wang et al., 2014), restore serum IgA and IgG levels in elderly mice with protein energy malnutrition (PEM), and contribute to the recovery of the immune system in immunocompromised hosts (Kaburagi et al., 2007). Additionally, *L. johnsonii* BS15 supplementation can prevent subclinical necrotizing enteritis (SNE) in broilers by improving immune related blood parameters and enhancing intestinal immunity (Wang et al., 2018). Consistent with previous findings, *L. johnsonii* L29 supplementation significantly increased serum IgG, IgM, and IgA levels in weaned calves in the present study, indicating that *L. johnsonii* L29 has a promoting effect on enhancing the immunity of weaned calves. Similarly, some studies have shown that *L. reuteri* can significantly increase serum IgG and IgA levels and enhance immune response in mice. In the present study, *L. reuteri* L81 supplementation significantly increased serum IgG and IgA levels at T2 and T3 compared with the Ctrl group. Moreover, supplementation with *L. reuteri* L81 and *L. johnsonii* L29 mixture significantly improved serum IgG, IgM, and IgA levels in calves at T3, indicating that supplementation with *L. reuteri* L81, *L. johnsonii* L29, or a combination of both can alleviate weaning-induced diarrhea by increasing calf immunoglobulin levels and enhancing immunity against viruses and bacteria.

SOD activity indirectly reflects the ability of the body to scavenge free oxygen radicals. Studies have shown that *L. reuteri* supplementation can significantly improve SOD activity in piglets (Yang et al., 2019). Similarly, oral administration of *L. reuteri* significantly increased SOD activity and decreased MDA levels in the intestinal tissue of mice with necrotizing enterocolitis (Tang et al., 2019). In the present study, probiotics supplementation significantly increased SOD activity at T1 and T3 compared with the Ctrl group, which was consistent with previous findings, indicating that probiotics can increase the activity of antioxidant enzymes in calves.

Weaning stress can damage the immune system and alter the production of inflammatory cytokines (Kim et al., 2012; Gurung et al., 2021), making the body extremely susceptible to infection by various pathogens. Studies have shown that some *L. reuteri* strains (including GMNL-263) can reduce the production of proinflammatory cytokines, such as TNF-α and IL-6, in mice fed a high fat diet (Hsieh et al., 2016). Additionally, *L. reuteri* significantly suppressed sodium dextran sulfate-induced expression of TNF-α (Hou et al., 2018). Consistent with previous findings, *L. reuteri* L81 supplementation significantly reduced serum IL-6 level at T3 compared with the Ctrl group in the present study. IL-10 is an anti-inflammatory cytokine that inhibits the expression of several pro-inflammatory cytokines and cell surface antigens, and improves immunopathology (Couper et al., 2008). In the present study, *L. johnsonii* L29 supplementation significantly increased serum level of IL-10 compared with the Ctrl group. Additionally, supplementation with *L. reuteri* L81 and a mixture of *L. johnsonii* L29 and *L. reuteri* L81 significantly increased serum IL-10 levels, which was consistent with previous findings that probiotics can bind to intestinal mucosal epithelial cells *via* toll-like receptors and mediate cytokine secretion to regulate the expression of immune T cells and the production of IL-10 to enhance the body’s immunity (Carolina et al., 2019). *L. reuteri* L81 and *L. johnsonii* L29 have been speculated to alleviate inflammatory reaction in the body, improve intestinal mucosal barrier function, and reduce the occurrence of diarrhea by stimulating the anti-inflammatory cytokine IL-10.

Factors, such as stress and autoimmune system dysfunction, have been shown to damage mucosal barrier function and alter intestinal wall permeability (Wang et al., 2016). ET, D-LA, and DAO can enter blood circulation *via* the damaged intestinal mucosa, making them important markers for evaluating intestinal mucosal barrier function (Kuang et al., 2020; He et al., 2022). In the present study, *L. johnsonii* L29 supplementation inhibited the leakage of D-LA and DAO into the peripheral blood and suppressed serum ET levels compared with the Ctrl group, indicating that *L. johnsonii* L29 had a protective effect on intestinal mucosal integrity. Similarly, *L. reuteri* L81 supplementation significantly reduced serum DAO levels, and serum concentration of D-LA at T1 and T2. *L. reuteri* has been speculated to alleviate weaning diarrhea by reducing intestinal mucosal damage. Additionally, *L. reuteri* L81supplementation significantly reduced serum levels of ET, indicating that *L. reuteri* can inhibit the growth of harmful intestinal bacteria and reduce the production of intestinal toxins. Similarly, supplementation with a mixture of *L. reuteri* L81 and *L. johnsonii* L29 decreased the serum levels of ET, D-LA, and DAO in weaned calves at different time points compared with the Ctrl group, indicating that the probiotics reduced intestinal mucosal permeability, improved mucosal barrier function, and reduced the leakage of endotoxins into blood circulation, thereby relieving weaning diarrhea in calves. In our research, we found that compared with *L. johnsonii* L29, under the same nutritional and cultural conditions, *L. reuteri* L81 has faster growth rate, shorter passage and preparation cycle, and lower economic and time costs for large-scale cultivation. Considering economic cost factors, it is more suitable for the preparation and production of probiotic formulations and is convenient for later promotion and application. Therefore, in the subsequent experiments, just *L. reuteri* L81 was studied.

### 4.4. Intestinal morphology

The villus height, crypt depth, and the ratio of villus height to crypt depth are major indicators in evaluating the integrity and digestive ability of the small intestine. When calves are weaned, due to the imperfect digestive system of calves and the change of dietary structure, it is easy to lead to the contraction of intestinal villi, the decrease of digestion and absorption enzyme activity of calves. We found that the supplementation of *L. reuteri* L81 increased the height of small intestinal villi, reduced the depth of crypts, and also had a positive effect on increasing the ratio between the two. The small intestinal villi mainly absorb amino acids, glucose, inorganic salts and other substances from the epithelial cells of the intestinal mucosa to provide nutrition for the body. The ratio of villus height to crypt depth can comprehensively reflect the digestion and absorption of the intestine. The larger the ratio, the more epithelial cells in the small intestine, the larger the absorption area of the small intestine, and the higher the absorption and utilization of nutrients. The results showed that the *L.reuteri* L81 increased the average daily gain of calves after weaning. It was speculated that this was related to the increase of villus height and the ratio of villus height / crypt depth, which was similar to the results of previous studies (Wang et al., 2020). In short, *L. reuteri* L81 enhances the integrity of intestinal tissue morphology in weaned calves, thereby promoting nutrient absorption and weight gain.

### 4.5. Expression of intestinal tight junction protein and cytokines related genes

There is a group of protein complexes composed of transmembrane proteins such as ZO-1, Occludin and Claudins between intestinal epithelial cells, which play a key regulatory role in maintaining intestinal barrier function and preventing intestinal mucosal injury (Günzel and Yu, 2013; Kuo et al., 2021). Bacteria and toxins can enter other tissues or organs through the damaged intestinal epithelial cell tight junction protein, thus causing bacterial and toxin translocation and many diseases. It has been reported that the supplementation of probiotics can enhance the intestinal physical barrier function by increasing the expression of intestinal epithelial tight junction protein. For example, *Lactobacillus rhamnosus* GG can protect the intestinal mucosa of rats from PTG induced damage by preventing the decrease of the expression of intercellular connexin (Orlando et al., 2018). *Lactobacillus reuteri* LR1 isolated from the feces of weaned piglets can increase the protein content of tight junction protein, *ZO-1* and *Occludin* in IPEC-1 cells infected by ETEC K88 (Yi et al., 2018). In this study, it was found that the gene expression of *ZO-1*, *Claudin-1* and *Occludin* in jejunum mucosa and *Claudin-1* in the ileum were significantly increased after weaning of Holstein calves supplemented with *L. reuteri* L81 before weaning, which was consistent with the results of previous studies. It shows that *L. reuteri* L81 plays an important role in enhancing intestinal barrier function and maintaining intestinal health. In addition, we also found that *L. reuteri* L81 significantly enhanced the gene expression of *TGF-β1* in jejunum and ileum mucosa, and significantly reduced the gene expression of *IL-8* in jejunum and *INF-γ* in ileum, indicating that this strain can regulate the immune performance of calves after weaning by enhancing the expression of anti-inflammatory factors and inhibiting the expression of pro-inflammatory factors, and reduce the intestinal mucosal damage caused by pathogenic bacteria, thereby alleviating the occurrence of weaning diarrhea in calves.

### 4.6. Enzyme activity in small intestine

The activity of digestive enzymes in the digestive tract is an important indicator of digestive function in animals. Na^+^/K^+^-ATPase is a transmembrane protein that plays an important role in maintaining Na^+^ −dependent transport vectors, such as sodium glucose co-transporter 1 (SGLT1)-mediated glucose transport (Manoharan et al., 2018). A previous study showed that children with ulcerative colitis with severe proctitis have significantly lower Na^+^/K^+^-ATPase activity than those with remission stage ulcerative colitis or with normal rectal mucosa, which may lead to impaired sodium transport-induced diarrhea (Ejderhamn et al., 1989). Additionally, *B. amyloliquefaciens* supplementation significantly increased intestinal Na^+^/K^+^-ATPase activity in piglets (Hu S. et al., 2018). Consistent with the previous findings, *L. reuteri* supplementation significantly increased the activity of Na^+^/K^+^-ATPase in the duodenum, jejunum, and ileum of weaned calves.

### 4.7. Regulation of intestinal microbiota

The intestinal microbiota plays crucial roles in the development, maturation, and homeostasis of the mucosal immune system (Hou et al., 2022), and an imbalance in the intestinal microbiota is associated with several diseases that cause inflammation of the intestinal tissue (Malmuthuge et al., 2015). Maintaining normal intestinal microbiota structure is necessary to maintain a healthy gut. There is increasing evidence that early intervention of intestinal microbiota during critical periods may be a promising method for improving intestinal microbial colonization (Xiang et al., 2020). Supplementation with probiotics improves early intestinal health and reduces intestinal diseases in calves (Aidy et al., 2013; Gensollen et al., 2016). This study conducted 16S rRNA and ITS gene amplicon sequencing on the small intestine contents of Ctrl and R groups of calves on the 21st day after weaning to analyze the effect of *L. reuteri* L81 on the composition and diversity of intestine microbiota in weaned calves.

Intestinal microbial diversity is related to the age, disease status and growth rate of calves. For example, intestinal microbial diversity was significantly lower in calves with diarrhea than in healthy calves (Oikonomou et al., 2013). Previous studies have shown that the supplementation of probiotics to calf diets can alter intestinal bacterial diversity and colony composition in the gastrointestinal tract (Wen et al., 2022). An abundant intestinal microbiota can resist pathogenic bacteria invasion; moreover, the higher the microbiota diversity the better is the nutritional status of the intestinal tract. In the present study, *L. reuteri* L81 supplementation increased the number of unique ASVs compared with the Ctrl group, with an increase in Chao1 and Shannon indices of intestinal microbiota, indicating that *Lactobacillus* enhanced the abundance and diversity of intestinal microbiota and improved disease resistance. Some studies have shown relatively low microbial diversity in the small intestine, similar microbial composition and diversity in the ileum and hindgut of Aohan Fine Wool Sheep, and significantly lower intestinal microbial diversity and abundance in the foregut than in the hindgut (Ma et al., 2022). In the present study, the number of unique ASVs and Chao1 and Shannon indices was significantly higher in the ileum than in the duodenum and jejunum, indicating a higher bacterial diversity and abundance in the ileum than in the duodenum and jejunum, which was consistent with previous findings (Myer et al., 2016; Freetly et al., 2020). However, there was no significant difference in Simpson index among the groups, indicating that *L. reuteri* L81 supplementation did not significantly affect bacterial uniformity in each intestinal segment. Additionally, NMDS analysis showed significant separation of microbial composition in the ileum, duodenum, and jejunum of calves with supplemented *L. reuteri* L81, indicating a certain difference in microbial community composition between the ileum and other intestinal regions, which is consistent with the research results of Chinese Mongolian sheep. NMDS analysis showed that there was a significant separation in microorganism composition in the ileum, duodenum, and jejunum of calves fed *Lactobacillus*-supplemented diet, indicating that there was a certain difference in the composition of microbial communities in the ileum and other intestinal regions, which was consistent with findings in Chinese Mongolian sheep (Zeng et al., 2017).

High-throughput sequencing showed that *Firmicutes*, *Bacteroides*, and *Actinobacteria* were the dominant bacteria in the small intestine of weaned calves. *Firmicutes* can participate in energy absorption in the intestine (Tap et al., 2010), and *Firmicutes* have been shown to be the dominant flora in the small intestine of ruminant and monogastric animals (Costea et al., 2017; Liu et al., 2019). The small intestinal microbial community is mainly composed of rapidly growing facultative anaerobic bacteria that can tolerate the combined effects of bile acids and antibiotics and effectively compete with the host and other bacteria for simple carbohydrates. Bile acids secreted by the proximal bile duct can inhibit the growth of *Bacteroides* spp. (Donaldson et al., 2016), which was confirmed by the relatively low abundance of *Bacteroidetes* in the duodenum and jejunum of calves in the present study, regardless of whether *L. reuteri* L81 was added or not.

At the genus level, there were similarities and differences in the dominant bacterial genera in various segments of the small intestine, which may be related to the physiological function of each intestinal segment. Specifically, *L. reuteri* L81 supplementation decreased the relative abundance of *Dialister* in the various segments of the small intestine. Among the human intestinal microbiota, *Dialister* has one of the lowest average abundances of core bacteria, but its role in the microbiota is poorly understood. However, the low abundance of *Dialister* has been shown to be related to Crohn’s disease (Joossens et al., 2011). *L. acidophilus* NCFM strain and cellobiose symbiotic bacteria supplementation for three weeks increased the relative abundance of *Bifidobacterium*, *Collinsella*, and *Eubacterium*, and decreased the relative abundance of *Dialister*, which is similar to the results of the present study. *Bifidobacteria* can improve the intestinal epithelial barrier function in animals by inhibiting the secretion of pro-inflammatory factors and improving the expression of intestinal tight junction proteins (Ling et al., 2016; Dong et al., 2022). An in-depth study of *Lactobacillus* showed that this bacterium could maintain the intestinal epithelial barrier function by inhibiting intestinal epithelial cell apoptosis, upregulating the expression of intestinal tight junction proteins, and improving the integrity of the intestinal mucosa (Hu S. et al., 2018; Zhou et al., 2022). *Oscillospira* is a genus of bacteria capable of producing short-chain fatty acids, such as butyric acid, which has positive effects in certain specific diseases and is considered a candidate for the next generation of probiotics (Yang et al., 2021). Several factors, including probiotics and prebiotics, promote the enrichment of *Oscillospira* spp. In the present study, *L. reuteri* L81 supplementation enriched *Bifidobacterium*, *Lactobacillus*, and *Oscillospira* in the duodenum, jejunum, and ileum, indicating that *L. reuteri* L81 can improve the intestinal barrier function of calves by promoting the relative abundance of probiotics, thereby alleviating weaning diarrhea. Several studies have shown that *Collinsella* and *Bifidobacterium* can modify host bile acids and regulate the virulence of intestinal pathogens (Elisha et al., 2017). *Bacteroides* and *Odoribacter* play important roles in maintaining the balance between intestinal microbiota and intestinal barrier function (Li et al., 2012). The enrichment of these bacteria in the ileum plays a positive role in maintaining the intestinal health in weaned calves. Additionally, *L. reuteri* L81 supplementation enriched fungi in the jejunum and ileum, indicating that the addition of this bacterium also had an impact on the composition of the fungal. For example, the *Thermomycetes* was enriched in the jejunum and ileum of the *L. reuteri* L81 group. Research has shown that *Thermomyces* is an excellent hemicellulose degrading bacterium that secretes xylanase (Lu et al., 2022). Increased serum triglyceride concentration and metabolic biomarkers in mice, including the deposition of hepatic lipids, weight gain, correlate with increased abundance of the fungal *Thermomyces* and decreased *Saccharomyces* (Mims et al., 2021). Research suggests that intestinal fungi are related to host health and disease; however, the role of fungi in intestinal health requires further study.

## 5. Conclusion

In our previous study, fecal microbial transplantation was carried out with Xinjiang yak as donor and Holstein calf as recipient. In this research, two strains of *Lactobacillus* (*L. reuteri* L81and *L. johnsonii* L29) isolated from the feces of Holstein calves after low concentration fecal microbiota transplantation had a positive effect on the growth performance, immune and antioxidant capacity in weaned calves, and alleviated weaning diarrhea by reducing intestinal permeability. Supplementation of *L. reuteri* L81 before weaning significantly reduced the ileal crypt depth and increased the ratio of villus height to crypt depth of ileal after weaning. It significantly up-regulated gene expression of tight junction proteins *ZO-1*, *Claudin-1*, *Occludin* and cytokine *TGF-β1* in jejunum mucosa, and increased gene expression of *Claudin-1* and *TGF-β1* in the ileum mucosa, reduced gene expression of pro-inflammatory factor *IL-8* in the jejunal mucosa and *INF- γ* in the ileal mucosa. *L. reuteri* L81 also significantly increased the Na^+^- K^+^- ATPase activity in the small intestine of weaned calves. In addition, it promotes the enrichment of beneficial bacteria *Bifidobacterium*, *Lactobacillus*, and *Oscillospira* in the small intestine. To sum up, *L. reuteri* L81 can effectively improve the intestinal tissue morphology of calves after weaning, enhance the intestinal epithelial barrier function, alleviate stress reactions and intestinal damage caused by early weaning, and help enhance the resistance of calves to weaning diarrhea.

## Data availability statement

The datasets presented in this study can be found in online repositories. The names of the repository/repositories and accession number(s) can be found in the article/Supplementary material.

## Ethics statement

The animal study was approved by Bioethics Committee of Shihezi University. The study was conducted in accordance with the local legislation and institutional requirements.

## Author contributions

WZ and YL designed the study. RL contributed to managing animals and sample collection. XL and YL contributed to the writing of the manuscript and the preparation of the first draft. CC, CN, YW, and JN have contributed to the comprehensive revision and editing of the manuscript. All authors contributed to the article and approved the submitted version.

## Funding

This work was supported by the Key Scientific and Technological Research Project in Key Fields of Xinjiang Production and Construction Corps (No. 2018AB041) and the Major Science and Technology Project of Xinjiang Production and Construction Corps (No. 2021AA004).

## Conflict of interest

RL was employed by Xinjiang Tianshan Junken Animal Husbandry Co., Ltd.

The remaining authors declare that the research was conducted in the absence of any commercial or financial relationships that could be construed as a potential conflict of interest.

## Publisher’s note

All claims expressed in this article are solely those of the authors and do not necessarily represent those of their affiliated organizations, or those of the publisher, the editors and the reviewers. Any product that may be evaluated in this article, or claim that may be made by its manufacturer, is not guaranteed or endorsed by the publisher.

## Supplementary material

The Supplementary material for this article can be found online at: https://www.frontiersin.org/articles/10.3389/fmicb.2023.1249628/full#supplementary-material

## References

[ref1] Aidy S. E. Hooiveld G. Tremaroli V. Bäckhed F. Kleerebezem M. (2013). The gut microbiota and mucosal homeostasis: colonized at birth or at adulthood, does it matter? Gut Microbes 4, 118–124. doi: 10.4161/gmic.23362, PMID: 23333858 PMC3595071

[ref2] Aoudia N. Rieu A. Briandet R. Deschamps J. Chluba J. Jego G.. (2016). Biofilms of Lactobacillus plantarum and *Lactobacillus fermentum*: effect on stress responses, antagonistic effects on pathogen growth and immunomodulatory properties. Food Microbiol. 53, 51–59. doi: 10.1016/j.fm.2015.04.009, PMID: 26611169

[ref3] Brand P. Gobeli S. Perreten V. (2017). Pathotyping and antibiotic resistance of porcine enterovirulent *Escherichia coli* strains from Switzerland (2014-2015). Schweiz. Arch. Tierheilkd. 159, 373–380. doi: 10.17236/sat00120, PMID: 28703707

[ref4] Brown K. Uwiera R. Kalmokoff M. L. Brooks S. Inglis G. D. (2017). Antimicrobial growth promoter use in livestock: a requirement to understand their modes of action to develop effective alternatives. Int. J. Antimicrob. Agents 49, 12–24. doi: 10.1016/j.ijantimicag.2016.08.006, PMID: 27717740

[ref5] Carolina M. G. Silvia I. C. María J. Lemme D. Eva V. Gabriela P. (2019). Beneficial effects of probiotic consumption on the immune system. Ann. Nutr. Metab. 74, 115–124. doi: 10.1159/000496426 30673668

[ref6] Clark E. O. Deller A. N. Harrelson P. Harrelson F. W. (2016). Effects of two-stage weaning duration on beef cattle growth and vocalizations. J. Anim. Sci. 95:58. doi: 10.2527/ssasas2017.0118

[ref7] Costea P. I. Hildebrand F. Arumugam M. Bäckhed F. Blaser M. J. Bushman F. D.. (2017). Enterotypes in the landscape of gut microbial community composition. Nat. Microbiol. 3, 8–16. doi: 10.1038/s41564-017-0072-8, PMID: 29255284 PMC5832044

[ref8] Couper K. N. Blount D. G. Riley E. M. (2008). IL-10: the master regulator of immunity to infection. J. Immunol. 180, 5771–5777. doi: 10.4049/jimmunol.180.9.5771 18424693

[ref9] David R. C. Patricia R. M. Abelardo M. Miguel G. De L. Nuria S. (2016). Intestinal short chain fatty acids and their link with diet and human health. Front. Microbiol. 7:185. doi: 10.3389/fmicb.2016.00185, PMID: 26925050 PMC4756104

[ref10] Dell’Anno M. Callegari M. L. Reggi S. Caprarulo V. Giromini C. Spalletta A.. (2021). Lactobacillus plantarum and *Lactobacillus reuteri* as functional feed additives to prevent diarrhoea in weaned piglets. Animals 11:1766. doi: 10.3390/ani11061766, PMID: 34204784 PMC8231520

[ref11] Deng Z. Dai T. Zhang W. Zhu J. Luo X. A.-O. Fu D.. (2020). Glyceraldehyde-3-phosphate dehydrogenase increases the adhesion of *Lactobacillus reuteri* to host mucin to enhance probiotic effects. Int. J. Mol. Sci. 21:9756. doi: 10.3390/ijms21249756, PMID: 33371288 PMC7766874

[ref12] Donaldson G. P. Lee S. M. Mazmanian S. K. (2016). Gut biogeography of the bacterial microbiota. Nat. Rev. Microbiol. 14, 20–32. doi: 10.1038/nrmicro3552, PMID: 26499895 PMC4837114

[ref13] Dong J. Ping L. Cao T. Sun L. Liu D. Wang S.. (2022). Immunomodulatory effects of the *Bifidobacterium longum* BL-10 on lipopolysaccharide-induced intestinal mucosal immune injury. Front. Immunol. 13:947755. doi: 10.3389/fimmu.2022.947755, PMID: 36091059 PMC9450040

[ref14] Ejderhamn J. Finkel Y. Strandvik B. (1989). Na, K-ATPase activity in rectal mucosa of children with ulcerative colitis and Crohn's disease. Scand. J. Gastroenterol. 24, 1121–1125. doi: 10.3109/00365528909089265, PMID: 2556782

[ref15] Elisha I. L. Botha F. S. McGaw L. J. Eloff J. N. (2017). The antibacterial activity of extracts of nine plant species with good activity against *Escherichia coli* against five other bacteria and cytotoxicity of extracts. BMC Complement. Altern. Med. 17:133. doi: 10.1186/s12906-017-1645-z, PMID: 28241818 PMC5329917

[ref16] Fan P. Kim M. Liu G. Zhai Y. Liu T. Driver J. D.. (2021). The gut microbiota of newborn calves and influence of potential probiotics on reducing diarrheic disease by inhibition ofpathogen colonization. Front. Microbiol. 12:772863. doi: 10.3389/fmicb.2021.772863, PMID: 34745079 PMC8567051

[ref17] Freetly H. C. Aaron D. Lindholm-Perry A. K. Thallman R. M. Keele J. W. Foote A. P.. (2020). Digestive tract microbiota of beef cattle that differed in feed efficiency. J. Anim. Sci. 98:skaa 008. doi: 10.1093/jas/skaa008, PMID: 31930312 PMC7297442

[ref18] Gensollen T. Iyer S. S. Kasper D. L. Blumberg R. S. (2016). How colonization by microbiota in early life shapes the immune system. Science 352, 539–544. doi: 10.1126/science.aad9378, PMID: 27126036 PMC5050524

[ref19] Günzel D. Yu A. S. L. (2013). Claudins and the modulation of tight junction permeability. Physiol. Rev. 93, 525–569. doi: 10.1152/physrev.00019.2012, PMID: 23589827 PMC3768107

[ref20] Gurung R. Abrahamsen F. W. Mullenix K. K. Abdela W. McElhenney W. Gurung N. (2021). Evaluation of KemTRACE (R) chromium on animal performance and immune system of weaned beef calves. J. Anim. Sci. 99, 11–12. doi: 10.1093/jas/skab096.019

[ref21] Han L. Azad M. A. K. Huang P. Wang W. Zhang W. Blachier F.. (2022). Maternal supplementation with different probiotic mixture from late pregnancy to day 21 postpartum: consequences for litter size, plasma and colostrum parameters, and fecal microbiota and metabolites in sows. Front. Vet. Sci. 9:726276. doi: 10.3389/fvets.2022.726276, PMID: 35211537 PMC8860973

[ref22] He L. Wang C. Simujide H. Aricha H. Zhang J. Liu B.. (2022). Effect of early pathogenic escherichia coli infection on the intestinal barrier and immune function in newborn calves. Front. Cell. Infect. Microbiol. 12:818276. doi: 10.3389/fcimb.2022.818276, PMID: 35265533 PMC8900010

[ref23] He T. Zhu Y. Yu J. Xia B. Liu X. Yang G.. (2019). *Lactobacillus johnsonii* L531 reduces pathogen load and helps maintain short-chain fatty acid levels in the intestines of pigs challenged with salmonella enterica infantis. Vet. Microbiol. 230, 187–194. doi: 10.1016/j.vetmic.2019.02.003, PMID: 30827387

[ref24] Hill C. Guarner F. Reid G. Gibson G. R. Merenstein D. J. Pot B.. (2014). The international scientific association for probiotics and prebiotics consensus statement on the scope and appropriate use of the term probiotic. Nat. Rev. Gastroenterol. Hepatol. 11, 506–514. doi: 10.1038/nrgastro.2014.66, PMID: 24912386

[ref25] Hong W. (2016). Effect of Lactobacillus on growth performance and mucosal immunity in weaned piglets. Master's thesis. Guangzhou (Guangdong Province): South China Agricultural University.

[ref26] Hou K. Wu Z. Chen X. Wang J. Zhang D. Xiao C.. (2022). Microbiota in health and diseases. Signal Transduct. Target. Ther. 7:135. doi: 10.1038/s41392-022-00974-4, PMID: 35461318 PMC9034083

[ref27] Hou Q. Ye L. Liu H. Huang L. Yang Q. Turner J. R.. (2018). Lactobacillus accelerates ISCs regeneration to protect the integrity of intestinal mucosa through activation of STAT3 signaling pathway induced by LPLs secretion of IL-22. Cell Death Differ. 25, 1657–1670. doi: 10.1038/s41418-018-0070-2, PMID: 29459771 PMC6143595

[ref28] Hou C. Zeng X. Yang F. Liu H. Qiao S. (2015). Study and use of the probiotic *Lactobacillus reuteri* in pigs: a review. J. Anim. Sci. Biotechnol. 6, 14–33. doi: 10.1186/s40104-015-0014-3, PMID: 25954504 PMC4423586

[ref29] Hsieh F. Lan C. E. Huang T. Chen K. Chai C. Chen W.. (2016). Heat-killed and live *Lactobacillus reuteri* GMNL-263 exhibit similar effects on improving metabolic functions in high-fat diet-induced obese rats. Food Funct. 7, 2374–2388. doi: 10.1039/c5fo01396h, PMID: 27163114

[ref30] Hu S. Cao X. Wu Y. Mei X. Xu H. Wang Y.. (2018). Effects of probiotic Bacillus as an alternative of antibiotics on digestive enzymes activity and intestinal integrity of piglets. Front. Microbiol. 9:2427. doi: 10.3389/fmicb.2018.02427 30405544 PMC6204369

[ref31] Hu J. Chen L. Zheng W. Shi M. Liu L. Xie C.. (2018). *Lactobacillus frumenti* facilitates intestinal epithelial barrier function maintenance in early-weaned piglets. Front. Microbiol. 9:897. doi: 10.3389/fmicb.2018.00897, PMID: 29867808 PMC5958209

[ref32] Joossens M. Huys G. Cnockaert M. Preter V. D. Vermeire S. (2011). Dysbiosis of the faecal microbiota in patients with Crohn's disease and their unaffected relatives. Gut 60, 631–637. doi: 10.1136/gut.2010.223263, PMID: 21209126

[ref33] Kaburagi T. Yamano T. Fukushima Y. Yoshino H. Mito N. Sato K. (2007). Effect of *Lactobacillus johnsonii* La1 on immune function and serum albumin in aged and malnourished aged mice. Nutrition 23, 342–350. doi: 10.1016/j.nut.2007.02.001, PMID: 17367996

[ref34] Kelsey A. J. Colpoys J. D. (2018). Effects of dietary probiotics on beef cattle performance and stress. J. Vet. Behav. 27, 8–14. doi: 10.1016/j.jveb.2018.05.010

[ref35] Kim M. H. Yun C. H. Lee C. H. Ha J. K. (2012). The effects of fermented soybean meal on immunophysiological and stress-related parameters in Holstein calves after weaning. J. Dairy Sci. 95, 5203–5212. doi: 10.3168/jds.2012-5317, PMID: 22916926

[ref36] Kotnik V. Zaletel J. (2011). Complement in diabetic nephropathy. Mol. Immunol. 48, 1719–1720. doi: 10.1016/j.molimm.2011.06.391

[ref37] Kuang J. H. Huang Y. Y. Hu J. S. Yu J. J. Zhou Q. Y. Liu D. M. (2020). Exopolysaccharides from *Bacillus amyloliquefaciens* DMBA-K4 ameliorate dextran sodium sulfate-induced colitis via gut microbiota modulation. J. Funct. Foods 75:104212. doi: 10.1016/j.jff.2020.104212

[ref38] Kuo W. Zuo L. Odenwald M. A. Madha S. Singh G. Gurniak C. B.. (2021). The tight junction protein zo-1 is dispensable for barrier function but critical for effective mucosal repair. Gastroenterology 161, 1924–1939. doi: 10.1053/j.gastro.2021.08.047, PMID: 34478742 PMC8605999

[ref39] Laxminarayan R. Duse A. Wattal C. Zaidi A. K. M. Wertheim H. F. L. Sumpradit N.. (2013). Antibiotic resistance-the need for global solutions. Lancet Infect. Dis. 13, 1057–1098. doi: 10.1016/S1473-3099(13)70318-9, PMID: 24252483

[ref40] Li R. W. Connor E. E. Li C. Vi R. L. B. Sparks M. E. (2012). Characterization of the rumen microbiota of pre-ruminant calves using metagenomic tools. Environ. Microbiol. 14, 129–139. doi: 10.1111/j.1462-2920.2011.02543.x 21906219

[ref41] Li Y. Li X. Wu Y. Zhang W. (2022). Effects of fecal microbiota transplantation from yaks on weaning diarrhea, fecal microbiota composition, microbial network structure and functional pathways in Chinese Holstein calves. Front. Microbiol. 13:898505. doi: 10.3389/fmicb.2022.898505, PMID: 36212876 PMC9537452

[ref42] Ling X. Linglong P. Weixia D. Hong W. (2016). Protective effects of Bifidobacterium on intestinal barrier function in LPS-induced enterocyte barrier injury of Caco-2 monolayers and in a rat NEC model. PLoS One 11:e0161635. doi: 10.1371/journal.pone.0161635, PMID: 27551722 PMC4995054

[ref43] Liu H. Roos S. Jonsson H. Ahl D. Dicksved J. Lindberg J. E.. (2015). Effects of Lactobacillus johnsonii and *Lactobacillus reuteri* on gut barrier function and heat shock proteins in intestinal porcine epithelial cells. Physiol. Rep. 3:e12355. doi: 10.14814/phy2.12355, PMID: 25847917 PMC4425961

[ref44] Liu J. Taft D. H. Maldonado-Gomez M. X. Johnson D. Treiber M. L. Lemay D. G.. (2019). The fecal resistome of dairy cattle is associated with diet during nursing. Nat. Commun. 10:4406. doi: 10.1038/s41467-019-12111-x, PMID: 31562300 PMC6765000

[ref45] Lu J. Wang J. Gao Q. Liu Z. Chen Z. Huang Q.. (2021). Effects of different probiotics on growth performance, serum biochemical indexes and fecal microflora of weaned goats. Chin. J. Anim. Nutr. 33, 2752–2764. doi: 10.3969/j.issn.1006-267x.2021.05.034

[ref46] Lu X. Yang Y. Hong C. Zhu W. Yao Y. Zhu F.. (2022). Optimization of vegetable waste composting and the exploration of microbial mechanisms related to fungal communities during composting. J. Environ. Manag. 319:115694. doi: 10.1016/j.jenvman.2022.115694 35841778

[ref47] Ma Y. Deng X. Yang X. Wang J. Li T. Hua G.. (2022). Characteristics of bacterial microbiota in different intestinal segments of Aohan fine-wool sheep. Front. Microbiol. 13:874536. doi: 10.3389/fmicb.2022.874536 35572716 PMC9097873

[ref48] Malmuthuge N. Griebel P. J. Guan L. L. (2015). The gut microbiome and its potential role in the development and function of newborn calf gastrointestinal tract. Front. Vet. Sci. 2:36. doi: 10.3389/fvets.2015.00036, PMID: 26664965 PMC4672224

[ref49] Manoharan P. Sundaram S. Singh S. Sundaram U. (2018). Inducible nitric oxide regulates brush border membrane na-glucose co-transport, but not na:h exchange via p38 map kinase in intestinal epithelial cells. Cells 7:111. doi: 10.3390/cells7080111, PMID: 30126234 PMC6115905

[ref50] Mcguirk S. M. (2008). Disease management of dairy calves and heifers. Vet. Clin. North Am. Food Anim. Pract. 24, 139–153. doi: 10.1016/j.cvfa.2007.10.003, PMID: 18299036 PMC7135781

[ref51] Mims T. S. Abdallah Q. A. Stewart J. D. Watts S. P. White C. T. Rousselle T. V.. (2021). The gut mycobiome of healthy mice is shaped by the environment and correlates with metabolic outcomes in response to diet. Commun. Biol. 4:281. doi: 10.1038/s42003-021-01820-z 33674757 PMC7935979

[ref52] Myer P. R. Wells J. E. Smith T. Kuehn L. A. Freetly H. C. (2016). Analysis of the gut bacterial communities in beef cattle and their association with feed intake, growth, and efficiency. J. Anim. Sci. 95, 3215–3224. doi: 10.2527/jas.2016.1059, PMID: 28727105

[ref53] Oikonomou G. Teixeira A. G. Foditsch C. Bicalho M. L. Machado V. S. Bicalho R. C.. (2013). Fecal microbial diversity in pre-weaned dairy calves as described by pyrosequencing of metagenomic 16S rDNA. Associations of faecalibacterium species with health and growth. PLoS One 8:e63157. doi: 10.1371/journal.pone.0063157, PMID: 23646192 PMC3639981

[ref54] Orlando A. Linsalata M. Bianco G. Notarnicola M. D’Attoma B. Scavo M. P.. (2018). *Lactobacillus rhamnosus* GG protects the epithelial barrier of wistar rats from the pepsin-trypsin-digested gliadin (PTG)-induced enteropathy. Nutrients 10:1698. doi: 10.3390/nu10111698, PMID: 30405050 PMC6265991

[ref55] Panahi Z. Kia V. Moghiman M. Doroud D. Paryan M. (2020). A fast and straightforward method for the purification of anti-immunoglobulin G (IgG) for coombs wright assay. J. Med. Microbiol. Infect. Dis. 8, 155–160. doi: 10.29252/JOMMID.8.4.155

[ref56] Pempek J. A. Watkins L. R. Bruner C. E. Habing G. (2019). A multisite, randomized field trial to evaluate the influence of lactoferrin on the morbidity and mortality of dairy calves with diarrhea. J. Dairy Sci. 102, 9259–9267. doi: 10.3168/jds.2019-16476, PMID: 31400894 PMC7094274

[ref57] Sweeney B. C. Rushen J. Weary D. M. Passillé A. M. D. (2010). Duration of weaning, starter intake, and weight gain of dairy calves fed large amounts of milk. J. Dairy Sci. 93, 148–152. doi: 10.3168/jds.2009-2427, PMID: 20059913

[ref58] Tan J. Cho H. Pholcharee T. Pereira L. S. Doumbo S. Doumtabe D.. (2021). Functional human IgA targets a conserved site on malaria sporozoites. Sci. Transl. Med. 13:eabg2344. doi: 10.1126/scitranslmed.abg2344, PMID: 34162751 PMC7611206

[ref59] Tang J. Guo C. Gong F. (2019). Protective effect of *Lactobacillus reuteri* against oxidative stress in neonatal mice with necrotizing enterocolitis. Nan Fang Yi Ke Da Xue Xue Bao 39, 1221–1226. doi: 10.12122/j.issn.1673-4254.2019.10.14, PMID: 31801706 PMC6867940

[ref60] Tap J. Mondot S. Levenez F. Pelletier E. Caron C. Furet J. P.. (2010). Towards the human intestinal microbiota phylogenetic core. Environ. Microbiol. 11, 2574–2584. doi: 10.1111/j.1462-2920.2009.01982.x, PMID: 19601958

[ref61] Wang H. Ni X. Xiaodan Q. Lei L. Xin J. Luo M.. (2018). Probiotic *Lactobacillus johnsonii* BS15 improves blood parameters related to immunity in broilers experimentally infected with subclinical necrotic enteritis. Front. Microbiol. 9:49. doi: 10.3389/fmicb.2018.00049, PMID: 29441047 PMC5797545

[ref62] Wang M. Wu H. Lu L. Jiang L. Yu Q. (2020). *Lactobacillus reuteri* promotes intestinal development and regulates mucosal immune function in newborn piglets. Front. Vet. Sci. 7:42. doi: 10.3389/fvets.2020.00042, PMID: 32118065 PMC7018766

[ref63] Wang L. Zhang J. Guo Z. Kwok L. Ma C. Zhang W.. (2014). Effect of oral consumption of probiotic Lactobacillus planatarum P-8 on fecal microbiota, SIgA, SCFAs, and TBAs of adults of different ages. Nutrition 30, 776–783.e1. doi: 10.1016/j.nut.2013.11.018, PMID: 24984992

[ref64] Wang S. Zhao S. Fang J. Ma D. Fu H. Li Z.. (2016). Role of stress and intestinal barrier dysfunction in inflammatory bowel disease. World Chin. J. Digestol. 24, 3248–3254. doi: 10.11569/wcjd.v24.i21.3248

[ref65] Wen J. Zhao W. Li J. Hu C. Zou X. Dong X. (2022). Dietary supplementation of chitosan oligosaccharide-clostridium butyricum synbiotic relieved early-weaned stress by improving intestinal health on pigeon squabs (columba livia). Front. Immunol. 13:926162. doi: 10.3389/fimmu.2022.926162 35844624 PMC9284028

[ref66] Wu H. Xie S. Miao J. Li Y. Wang Z. Wang M.. (2020). *Lactobacillus reuteri* maintains intestinal epithelial regeneration and repairs damaged intestinal mucosa. Gut Microbes 11, 997–1014. doi: 10.1080/19490976.2020.1734423, PMID: 32138622 PMC7524370

[ref67] Xiang Q. Wu X. Pan Y. Wang L. (2020). Early intervention using fecal microbiota transplantation combined with probioticsinfluence the growth performance, diarrhea, and intestinal barrier function of piglets. Appl. Sci. 10:568. doi: 10.3390/app10020568

[ref68] Xin J. Chai Z. Zhang C. Zhang Q. Zhu Y. Cao H.. (2019). Comparing the microbial community in four stomach of dairy cattle, yellow cattle and three yak herds in Qinghai-Tibetan plateau. Front. Microbiol. 10:1547. doi: 10.3389/fmicb.2019.01547, PMID: 31354656 PMC6636666

[ref69] Yang J. Li Y. Wen Z. Liu W. Meng L. Huang H. (2021). Oscillospira-a candidate for the next-generation probiotics. Gut Microbes 13:1987783. doi: 10.1080/19490976.2021.1987783, PMID: 34693878 PMC8547878

[ref70] Yang J. Wang C. Liu L. Zhang M. (2019). *Lactobacillus reuteri* KT260178 supplementation reduced morbidity of piglets through its targeted colonization, improvement of cecal microbiota profile, and immune functions. Probiotics Antimicrob. Proteins 12, 194–203. doi: 10.1007/s12602-019-9514-3, PMID: 30659502

[ref71] Yi H. Wang L. Xiong Y. Wang Z. Qiu Y. Wen X.. (2018). *Lactobacillus reuteri* LR1 improved expression of genes of tight junction proteins via the MLCK pathway in IPEC-1 cells during infection with Enterotoxigenic *Escherichia coli* K88. Mediat. Inflamm. 2018:6434910. doi: 10.1155/2018/6434910, PMID: 30210262 PMC6120278

[ref72] Zeng Y. Zeng D. Ni X. Zhu H. Jian P. Zhou Y.. (2017). Microbial community compositions in the gastrointestinal tract of chinese mongolian sheep using Illumina MiSeq sequencing revealed high microbial diversity. AMB Express 7:75. doi: 10.1186/s13568-017-0378-1, PMID: 28378284 PMC5380569

[ref73] Zhou Q. Wu F. Chen S. Cen P. Yang Q. Guan J.. (2022). *Lactobacillus reuteri* improves function of the intestinal barrier in rats with acute liver failure through Nrf-2/HO-1 pathway. Nutr. Food Sci. 99-100:111673. doi: 10.1016/j.nut.2022.111673, PMID: 35567844

