# Supplementary material for: Effects of two strains of Lactobacillus isolated from the feces of calves after fecal microbiota transplantation on growth performance, immune capacity, and intestinal barrier function of weaned calves
Source: Front Microbiol. 2023 Aug 31;14:1249628. doi: 10.3389/fmicb.2023.1249628 (PMC10505964; doi:10.3389/fmicb.2023.1249628)
Supplement: Supplementary file 1 [file Table_1.DOC]

Supplementary Table 1. Composition and nutrient levels of control diet (DM basis, %)

| Item | Starter |
| --- | --- |
| Corn | 55.20 |
| Soybean meal | 18.50 |
| Corn gluten meal | 10.00 |
| DGGS | 13 |
| Limestone | 1.80 |
| NaCl | 0.50 |
| Premix^1^ | 1.00 |
| Total | 100.00 |
| Chemical analysis of starter |  |
| Dry Matte (DM) | 91.14 |
| Crude Protein (CP) | 20.63 |
| Ether extract (EE) | 11.36 |
| Acid Detergent Fiber (ADF) | 16.06 |
| Neutral detergent fiber (NDF) | 34.01 |
| Crude ash (Ash) | 7.02 |
| Calcium | 1.47 |
| Phosphorus | 1.32 |
| Chemical analysis of Arrhenatherum elatius |  |
| Dry Matte (DM) | 95.65 |
| Crude Protein (CP) | 4.97 |
| Ether extract (EE) | 6.12 |
| Acid Detergent Fiber (ADF) | 32.63 |
| Neutral detergent fiber (NDF) | 57.89 |
| Crude ash (Ash) | 5.47 |
| Calcium | 0.86 |
| Phosphorus | 0.40 |

Supplementary Table 2. Primer sequences of intestinal tight junction protein and cytokine genes

| **Genes** | **Forward primer sequence 5’-3’** | **Reverse primer sequence 5’-3’** |
| --- | --- | --- |
| *Claudin-1* | GTTCTTCACACATACCCCGTC | ATCCTCTGCTTTTCCTGCTC |
| *Occuludin* | GGCAACACAGGCGAAAAT | TTCCTCTGCTTTTCCTGCTC |
| *ZO-1* | GGAGAGGTGTTCCGTGTCG | TCTGTTTTTGTTTGGGATGATG |
| *TLR4* | GGGCAGGGAAAGTCAACTAAAC | TCAATCAAAACGACCAAAAACAC |
| *INF-γ* | CCGAGCGTGGAGGATCATTGC | CCAACGAGGCACAGCAGGATG |
| *TGF-β1* | CGAGCCCTGGACACCAACTA | AGGCAGAAATTGGCATGGT |
| *IL-2* | CCTGAGCAGGATGGAGAATTAC | TCCAGAACATGCCGCAGAG |
| *IL-8* | ACACATTCCACACCTTTCCAC | ACCTTCTGCACCCACTTTTC |
